# The UKB envirome of depression: from interactions to synergistic effects

**DOI:** 10.1038/s41598-019-46001-5

**Published:** 2019-07-05

**Authors:** Gabor Hullam, Peter Antal, Peter Petschner, Xenia Gonda, Gyorgy Bagdy, Bill Deakin, Gabriella Juhasz

**Affiliations:** 1 Department of Measurement and Information Systems, Budapest University of Technology and Economics, Budapest, H-1117 Hungary; 2 MTA-SE Neuropsychopharmacology and Neurochemistry Research Group, Hungarian Academy of Sciences, Semmelweis University, Budapest, H-1089 Hungary; 3 Department of Pharmacodynamics, Faculty of Pharmacy, Semmelweis University, Budapest, H-1089 Hungary; 4 NAP2-SE New Antidepressant Target Research Group Semmelweis University, Budapest, H-1089 Hungary; 5 Department of Psychiatry and Psychotherapy, Semmelweis University, Budapest, Hungary; 6 Neuroscience and Psychiatry Unit, Division of Neuroscience and Experimental Psychology, University of Manchester and Manchester Academic Health Sciences Centre, Manchester, M13 9PL UK; 7 Greater Manchester Mental Health NHS Foundation Trust, Prestwich, Manchester UK; 8 SE-NAP2 Genetic Brain Imaging Migraine Research Group, Semmelweis University, Budapest, H-1089 Hungary

**Keywords:** Probabilistic data networks, Depression, Depression

## Abstract

Major depressive disorder is a result of the complex interplay between a large number of environmental and genetic factors but the comprehensive analysis of contributing environmental factors is still an open challenge. The primary aim of this work was to create a Bayesian dependency map of environmental factors of depression, including life stress, social and lifestyle factors, using the UK Biobank data to determine direct dependencies and to characterize mediating or interacting effects of other mental health, metabolic or pain conditions. As a complementary approach, we also investigated the non-linear, synergistic multi-factorial risk of the UKB envirome on depression using deep neural network architectures. Our results showed that a surprisingly small number of core factors mediate the effects of the envirome on lifetime depression: neuroticism, current depressive symptoms, parental depression, body fat, while life stress and household income have weak direct effects. Current depressive symptom showed strong or moderate direct relationships with life stress, pain conditions, falls, age, insomnia, weight change, satisfaction, confiding in someone, exercise, sports and Townsend index. In conclusion, the majority of envirome exerts their effects in a dynamic network via transitive, interactive and synergistic relationships explaining why environmental effects may be obscured in studies which consider them individually.

## Introduction

Major depressive disorder (MDD) is a complex illness which is the leading cause of disability in the working population that results in severe decrease in life quality and elevated risks concerning several diseases^1–3^. Its clinical definition is still evolving and MDD is frequently viewed as a clinical construct associated with a set of symptoms originating from potentially different biological processes^4^.

Despite of its complex etiology and uncertain clinical manifestations, MDD has a strong, heritable genetic background: additive genetic effects are attributed to explain approximately 40% of the variation of susceptibility to this disorder^5^. However, only a handful of genetic variants were found associated with MDD on a genome-wide significance level by earlier genome-wide association (GWA) studies^6^. Although a recent study significantly increased the number of hits, the identification of these variants required enormous data sets consisting of several hundred-thousands subjects^4^. This is in line with the common variant hypothesis postulating that a considerable proportion of genetic variants affecting the development of MDD have only weak effects, and thus, only the interaction of several factors could produce considerable increase in risk for MDD. This phenomenon was one of the possible causes of the lack of success of earlier GWA studies which were typically underpowered to detect factors with low effect size^7^.

Accumulating results led to the realization that MDD is a result of the interplay of a large number of environmental and genetic factors: environmental and lifestyle descriptors such as years of education, body mass index, negative life events and childhood adversities were found to be significant factors in (G × E) interactions with respect to MDD indicating potential moderating effects of these factors on genetic vulnerability^8–13^. In addition, neuroticism or current depressive symptoms, which show considerable genetic overlap with MDD^4^, may mediate these environmental effects^14,15^. Non-replicability of GWA studies was also linked to the lacking or inadequate representation of environmental factors, resulting that such studies did not take gene-environment interactions (G × E) into consideration. Recent effort to investigate gene-environment wide interaction (GEWI) suggests that these interactions are of potential importance^16^.

Despite the fundamental role of environmental factors in common diseases, their comprehensive, omic-like analysis is lacking, mainly due to the heterogeneity of these factors. In MDD research, for example, the effect of environmental factors are typically well-explored individually in association with given genetic variants, but current approaches still disregard complex multivariate relationships between environmental factors, excluding potential higher-order environment-environment (E × E) interactions, which may prove to be vital for the further research of such complex disorders as major depression. Furthermore, association tests and simple regression analyses do not provide information on the directness of relationships with major depression, that is whether the effect of an environmental factor is mediated by other variables or not. Distinguishing between direct and non-direct relationships is especially relevant when selected factors serve as environmental context (in G × E) for assessing the relevance of genetic variants. Environmental factors that do not affect the target directly can be less interpretable and clinically less useful contexts. Therefore, a detailed map of the dependency relationships of environmental factors related to major depression would enhance further research.

Probabilistic graphical models proved to be an essential tool to represent the structural and the quantitative aspects of a system of dependencies^17^. They allow computationally efficient, scalable approaches to explore sparse models^18^ and causal effects^19,20^ in the frequentist framework and also the fine mapping of weak dependencies in the Bayesian approach^21–23^. We utilized Bayesian networks in the Bayesian approach referred to as Bayesian multilevel analysis of relevance (BMLA) to support the systematic, consistent exploration of the complete hierarchy of higher-order interactions, even in the case of complex phenotypes^24^. The approach also characterizes the relevance of factors at global levels estimating posteriors for wide range of dependency and causal relations, which we previously applied to investigate multimorbidities of depression^25^.

In this paper we investigate the interdependencies of social, environmental, lifestyle, metabolic and mental health factors in lifetime depression using the UK Biobank resource (application No. 1602). Our goals are as follows: (1) construction of a Bayesian dependency map of the UKB envirome for lifetime depression, (2) characterization of the relationships in the envirome map based on their directness, interactions, and synergistic effects, and (3) investigation of the non-linear joint effect of the envirome on depression and also the predictive power of relevant sets of envirome variables. In order to achieve the former two goals we applied the BMLA method; while for the third goal we constructed and utilized deep neural networks.

## Results

**Figure 1:**
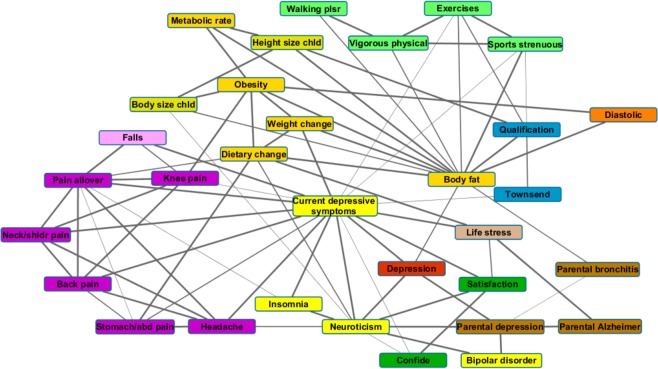
Bayesian map of the UKB envirome related to reported lifetime depression based on Bayesian relevance analysis. Nodes represent investigated variables, their coloring corresponds to the respective variable group as follows. (**A**) Mental health descriptors - yellow, (**B**) Social factors - dark green, (**C**) Childhood descriptors - lime, (**D**) Parental illnesses - brown, (**E**) Lifestyle and sports - light green, (**F**) Diet and metabolism - gold, (**G**) Blood pressure - orange, (**H**) Financial background and qualification - dark blue, (**I**) Pain - purple, (**J**) Life stress - light brown, (**K**) Falls - pink, and reported lifetime depression - red. An edge between two nodes represents a direct relationship, and its width is proportional to the Bayesian edge probability which takes into account both possible edge directions assuming an underlying Bayesian network. Edges with a probability lower than 0.5 are omitted.

In the following sections, we present the results of multiple analyses performed using specific tools. First, we provide a detailed description of the envirome map based on the variable dependency structure estimated by the BMLA method. Results are shown both as an undirected graph and as edge probabilities (Fig. 1). Second, we introduce the strong relevance measure provided by the BMLA method and compare relevant factors with respect to lifetime depression (reported by the participants and elaborated by trained research nurses) and probable depression diagnosis (derived from the Mental Health Questionnaire with the method described by Smith *et al*.^26^). Third, we analyze structural interactions revealed by the BMLA method, and perform parametric analysis using multivariate odds ratios. Fourth, we investigate synergistic effects which differ from structural interactions as all involved variables have an individual effect. Finally, we present results of deep neural networks assessing predictive power. In addition to comparing strongly associated variables in terms of predictive capabilities, we also investigate the predictive power of variable groups.

### The map of depression envirome

The primary aim of our study was to identify factors that may influence susceptibility to lifetime depression or the severity of its symptoms. This requires multiple analyses that investigate possible relationships qualitatively and quantitatively. We applied the BMLA method for structural analysis, which provides posteriors for arbitrary dependency, relevance and causal patterns based on the multivariate dependency structure learned from data^21,22,24^.

Figure 1 presents the network of variable relationships with respect to reported lifetime depression up to its second neighbors (sex and age and their connections are omitted for visibility purposes). An edge between variables *X* and *Y* represents that based on Bayesian relevance analysis a dependency relationship exists between *X* and *Y* with a probability not less than 0.5. Note that this edge probability takes into account both possible directions of a directed edge assuming an underlying Bayesian network structure representing dependencies.

One of the remarkable features of this graph is that reported lifetime depression has only a few direct relationships, namely current depressive symptoms, neuroticism, parental depression, and body fat percentage. All other factors shown on the graph are in a non-direct relationship with reported lifetime depression, most of them mediated by current depressive symptoms. Table 1 shows the probabilities of various relationship types such as direct and transitive relationships concerning lifetime depression and current depressive symptoms. A direct relationship between two variables *X* and *Y* means there are no intermediary variables between them, i.e., they are connected by a directed edge (*X* → *Y* or *Y* → *X*) in the graph representing dependency relationships. On the other hand, a transitive relationship between *X* and *Y* means that there are one or more intermediary variables (*Z*) in between such that there is a path formed between *X* and *Y* by directed edges in the dependency graph (*X* → *Z* → *Y* or *Y* → *Z* → *X*).

**Table 1:** The posterior probability of variable relationships with respect to reported lifetime depression and current depressive symptoms.

Variable	Lifetime depression		Current depressive symptoms		Variable	Lifetime depression		Current depressive symptoms	
	DIR	TRN	DIR	TRN		DIR	TRN	DIR	TRN
Age	0.00	0.80	0.999	0.001	Neuroticism	0.999	0.001	0.999	0.001
Alcohol intake	0.00	0.40	0.00	0.8	Obesity	0.00	0.60	0.20	0.80
Back pain	0.00	0.40	1.00	0.00	Pain allover	0.00	0.80	1.00	0.00
Body fat	0.80	0.00	0.00	1.00	Parental Alzheimer’s	0.00	0.80	0.00	1.00
Body size	0.00	0.40	0.00	1.00	Parental bronchitis	0.00	0.498	0.001	0.999
Breastfed	0.00	0.40	0.00	1.00	Parental cancer	0.00	0.80	0.00	0.999
Confide	0.00	0.20	0.60	0.40	Parental depression	0.999	0.00	0.00	1.00
Current depressive symptoms	0.999	0.001	—	—	Parental diabetes	0.00	0.60	0.00	1.00
Diastolic	0.00	0.60	0.00	1.00	Parental heart disease	0.00	0.60	0.00	1.00
Dietary change	0.00	0.20	0.20	0.80	Parental high bloodpressure	0.00	0.20	0.00	1.00
Exercises	0.00	0.40	0.60	0.40	Parental Parkinson’s	0.00	0.80	0.00	1.00
Facial pain	0.00	0.20	0.00	0.80	Parental stroke	0.00	0.80	0.00	1.00
Falls	0.00	0.40	1.00	0.00	Pulse	0.00	0.20	0.00	1.00
First intercourse	0.00	0.20	0.00	1.00	Qualification	0.00	0.20	0.00	1.00
Hand	0.00	0.20	0.00	1.00	Risk taking	0.00	0.20	0.00	1.00
Headache	0.00	0.80	1.00	0.00	Satisfaction	0.00	0.60	1.00	0.00
Heavy DIY	0.00	0.60	0.40	0.60	Sex	0.00	0.80	0.00	1.00
Height size	0.00	0.20	0.00	0.999	Social activity	0.00	0.40	0.20	0.80
Hip pain	0.00	0.475	0.474	0.326	Sports	0.00	0.60	0.60	0.40
Household income	0.20	0.00	0.40	0.60	Stomach/abdominal pain	0.00	0.201	0.80	0.20
Insomnia	0.00	0.00	0.999	0.00	Systolic	0.00	1.00	0.20	0.80
Knee pain	0.00	0.40	0.60	0.40	Tobacco smoking	0.00	0.20	0.20	0.80
Life stress	0.20	0.40	1.00	0.00	Townsend	0.00	0.60	0.60	0.40
Light DIY	0.00	0.20	0.00	1.00	Vigorous physical	0.00	0.40	0.40	0.60
Bipolar disorder	0.00	0.385	0.187	0.812	Visits	0.00	0.20	0.00	1.00
Maternal smoking	0.00	0.40	0.00	1.00	Walking	0.00	0.20	0.20	0.80
Metabolic rate	0.00	0.20	0.00	1.00	Walking physical	0.00	0.20	0.00	1.00
Moderate physical	0.00	0.20	0.00	1.00	Weight change	0.00	0.60	1.00	0.00

Displayed relationship types include direct relationship denoted as DIR, and transitive relationship denoted as TRN. Transitive relationship means that there are one or more other variables mediating the effect of a selected variable on the target variable.

#### Life stress and mental health factors

According to our results, the probability that recent negative life events of the past two years (denoted as Life stress) is in a direct relationship with lifetime depression is relatively low (*p*_*Dir*_ = 0.2), whereas the probability of a direct relationship with current depressive symptoms is high (*p*_*Dir*_ = 1.0). Conversely, the probability of a transitive relationship is relatively higher in the former case (*p*_*Trans*_ = 0.4), and low in the latter case (*p*_*Trans*_ = 0.0).

The neuroticism personality trait (denoted as Neuroticism) is in a direct relationship with both lifetime depression and current depressive symptoms with high probability (*p*_*Dir*_ = 0.999). In addition, it is directly related to parental depression and bipolar disorder. The sleep quality descriptor (denoted as Insomnia) is not related directly to lifetime depression, but it is in a direct relationship with current depressive symptoms (*p*_*Dir*_ = 0.999) and neuroticism (*p*_*Dir*_ = 0.999) with a high probability.

#### Social factors

Mental health related social factors such as being able to confide in someone (denoted as Confide), and the sense of satisfaction (Satisfaction) are only transitively connected to lifetime depression (*p*_*Trans*_ = 0.2 and 0.6 respectively) as no direct connection was detected. Regarding current depressive symptoms, the probability of a direct relationship with satisfaction is high (*p*_*Dir*_ = 1.0), and moderately high in case of Confide (*p*_*Dir*_ = 0.6).

#### Pain descriptors

Variables describing short or long-term presence of pain (i.e. Headache, Back pain, Stomach or abdominal pain, Neck or shoulder pain, Knee pain, and Pain allover) are not in a direct relationship with lifetime depression (*p*_*Dir*_ = 0.0). Instead, results indicate transitive relationships with lifetime depression such that most pain related variables are mediated by current depressive symptoms. For example, the probability of a direct relationship between neck or shoulder pain and lifetime depression is negligible (*p*_*Dir*_ = 0.0), whereas a transitive relationship is more probable having a moderately high probability (*p*_*Trans*_ = 0.629). In contrast to this, the probability of a direct relationship with current depressive symptoms is remarkably high (*p*_*Dir*_ = 1.0). In addition, the transitive relationship of headache with lifetime depression (*p*_*Trans*_ = 0.8) is not only mediated by current depressive symptoms (*p*_*Dir*_ = 1.0), but also mediated by neuroticism with which it has a direct relationship with high probability (*p*_*Dir*_ = 0.8).

#### Dietary change and metabolic descriptors

Variables related to dietary change and metabolism have multiple relations with lifetime depression and current depressive symptoms. Body fat percentage (Body fat), which is highly correlated with the obesity descriptor (Obesity), is in a direct relationship with lifetime depression with high probability (*p*_*Dir*_ = 0.8). In contrast with body fat percentage, obesity is not in a direct relationship with lifetime depression (*p*_*Dir*_ = 0.0), and the probability of direct connection with current depressive symptoms is also low (*p*_*Dir*_ = 0.2). On the other hand, a transitive relationship is more probable in both cases (*p*_*Trans*_ = 0.6 and 0.8 respectively). Furthermore, weight change is directly connected only to current depressive symptoms (*p*_*Dir*_ = 1.0). In addition, the metabolism descriptor (denoted as Metabolic rate) is not in a direct relationship with either lifetime depression or current depressive symptoms (*p*_*Dir*_ = 0.0). Similarly to the obesity descriptor, the variable indicating a substantial change in diet (Dietary change) is not in a direct relationship with lifetime depression (*p*_*Dir*_ = 0.0), and the probability of a transitive relationship with current depressive symptoms is higher (*p*_*Trans*_ = 0.8) than that of a direct (*p*_*Dir*_ = 0.2).

#### Sports and physical activity

Physical activity related variables such as strenuous sports, exercises, walking, vigorous physical activity, moderate physical activity are not in a direct relationship with lifetime depression, instead corresponding transitive relationships are of moderate probability (strenuous sports *p*_*Trans*_ = 0.6, exercises *p*_*Trans*_ = 0.4, vigorous physical activity *p*_*Trans*_ = 0.4, walking *p*_*Trans*_ = 0.2) mostly mediated by body fat. Concerning current depressive symptoms, the probability of direct relationships with physical activity descriptors is moderate (strenuous sports *p*_*Dir*_ = 0.6, exercises *p*_*Dir*_ = 0.6, vigorous physical activity *p*_*Dir*_ = 0.4, walking *p*_*Dir*_ = 0.2).

#### Financial background and qualification

Among the investigated socioeconomic status descriptors, the Townsend deprivation index (Townsend) is not connected directly to lifetime depression (*p*_*Dir*_ = 0.0), but there is a moderate probability of a transitive relationship (*p*_*Trans*_ = 0.6) mediated by current depressive symptoms. The probability of a direct relationship between household income and lifetime depression is relatively low (*p*_*Dir*_ = 0.2), although it plays a remarkable role in some of the interactions detailed later. Regarding current depressive symptoms, a transitive relationship with household income is more probable (*p*_*Trans*_ = 0.6) than a direct one (*p*_*Dir*_ = 0.4). Qualification (that is whether the subject has a college or university degree) is not connected directly to either lifetime depression (*p*_*Dir*_ = 0.001) or to current depressive symptoms (*p*_*Dir*_ = 0.0). However, the probability that there is a transitive relationship between current depressive symptoms and qualification is high (*p*_*Dir*_ = 1.0).

#### Parental illnesses

Parental depression is the only parental illness descriptor that is directly related to lifetime depression with high probability (*p*_*Dir*_ = 0.999), all other descriptors are only transitively related with various degrees of probability. Results also indicate a transitive relationship with current depressive symptoms with high probability for all such descriptors (*p*_*Trans*_ > 0.999).

#### Other factors

According to our results, alcohol intake and tobacco consumption are not in a direct relationship with lifetime depression (*p*_*Dir*_ = 0.0), the probability of a transitive relationship is also low in both cases (*p*_*Trans*_ = 0.4 and 0.2 respectively). However, both variables are in a transitive relationship with current depressive symptoms with high probability (*p*_*Trans*_ = 0.8).

Age and sex are both not directly connected to lifetime depression, although there is a high probability of a transitive relationship. Regarding current depressive symptoms, the probability of a direct relationship with age is high (*p*_*Dir*_ = 0.999), while sex is transitively related with high probability (*p*_*Trans*_ = 1). Among the investigated childhood descriptors none of the variables are in a direct relationship with lifetime depression, however it should be noted that childhood trauma items were not available for this analysis.

### Relevance of environmental factors

Identifying direct relationships is a major step towards discovering relevant factors, however relevance can be interpreted in multiple ways. Here we utilize the strong relevance concept according to which strongly relevant variables of a selected target variable consist of (1) direct relationships and (2) interaction terms that have a joint effect on the target involving another variable. This requires the analysis of relevant sets of variables with respect to the target variable. In a Bayesian structural approach, strong relevance (or relevance for short) of a variable is quantified by the posterior probability of the occurrence of the variable in possible models as a direct relationship or as an interaction term with respect to the target (see Strong relevance section of methods for details).

Table 2 shows posterior probabilities of strong relevance (*p*_*Rel*_) for relevant variables with respect to lifetime depression using a cutoff value of 0.2 including both direct and interaction type relations. These results indicate that in addition to the previously investigated direct relationships, i.e. current depressive symptoms (*p*_*Rel*_ = 1.0), neuroticism trait (*p*_*Rel*_ = 0.999), parental depression (*p*_*Rel*_ = 1.0), and body fat percentage (*p*_*Rel*_ = 0.8), there are several other variables that are relevant with respect to lifetime depression to some extent due to multivariate interactions. In other words, there are interaction terms forming multivariate interaction patterns involving lifetime depression. For example, sex, risk taking, parental Alzheimer’s disease and parental bronchitis are such factors that have a moderate probability of being interaction terms, and thus they can be considered as strongly relevant variables to a certain degree (*p*_*Rel*_ > 0.3). On the other hand, household income and life stress are in a direct relationship with lifetime depression with a low but non-negligible probability (*p*_*Rel*_ > 0.2) and consequently can be considered worthy of further investigation.

**Table 2:** Probability of strong relevance and dependency types with respect to lifetime depression.

Variable	Direct relation	Interaction term	Relevance
Current depressive symptoms	0.999	0.001	1.000
Parental depression	0.999	0.001	1.000
Bipolar disorder	0.000	0.999	0.999
Neuroticism	0.999	0.000	0.999
Body fat	0.800	0.000	0.800
Parental bronchitis	0.000	0.477	0.477
Parental Alzheimer’s	0.000	0.400	0.400
Sex	0.000	0.395	0.395
Risk taking	0.000	0.328	0.328
Maternal smoking	0.000	0.201	0.201
Moderate physical activity	0.000	0.200	0.200
Qualification	0.000	0.200	0.200
Age	0.000	0.200	0.200
Household income	0.200	0.000	0.200
Alcohol intake	0.000	0.200	0.200
Life stress	0.200	0.000	0.200
Exercises	0.000	0.200	0.200
Sports	0.000	0.200	0.200

Displayed relationship types include direct relations and interaction terms. Associated posterior probabilities reflect the likeliness that a variable is in a given type of relationship with lifetime depression. The probability of strong relevance is the sum of these probabilities.

In addition, we investigated relevant relationships with respect to probable depression diagnosis variables (single depressive episode, moderate depression, severe depression) created by Smith *et al*.^26^ and compared it to lifetime depression as a validation (see the Validation section of methods for details). Results indicate that the envirome map of lifetime depression is similar to that of probable depression diagnosis, such that it consists of similar patterns regarding current depressive symptoms, neuroticism, parental depression, and several moderately relevant factors. A notable difference between the two relationship maps is that while lifetime depression is directly connected only to body fat percentage among diet and metabolism related variables, in case of probable depression diagnosis this relationship is partially replaced by connections with obesity and dietary change.

### Environment-environment interactions

#### Structural interactions

In order to analyze interactions first we investigated strongly relevant sets (with respect to lifetime depression) provided by the applied BMLA method (see the methods section for details). These sets can also be called as structurally relevant sets of variables as they are based on the dependency structure of variables. Table 3 presents the top 4 most probable structurally relevant variable sets detailing the components of possible structural interaction patterns.

**Table 3:** Relationship types of variables within strongly relevant sets.

Relevant sets	Direct relations	Interaction terms	
1	Current depressive symptoms	Sex	
	Neuroticism	Risk taking	
	Parental depression	Bipolar disorder	
	Body fat		
2	Current depressive symptoms	Qualification	
	Neuroticism	Parental bronchitis	
	Parental depression	Bipolar disorder	
	Body fat		
3	Current depressive symptoms	Sports	Parental bronchitis
	Neuroticism	Exercises	Parental Alzheimer’s
	Parental depression	Moderate physical	Bipolar disorder
	Body fat	Age	
	Household income	Alcohol intake	
4	Current depressive symptoms	Maternal smoking	
	Neuroticism	Parental Alzheimer’s	
	Parental depression	Bipolar disorder	
	Life stress		

Generally, structural interaction patterns have at least one term that has an individual main effect (direct relationship) with respect to the target variable, while the other terms typically have minor or negligible effects individually. The key feature of interactions is the multivariate context, in which a particular set of variables have a considerable effect on the target variable. This context is provided either by the variable with the individual main effect or by additional variables.

**Figure 2:**
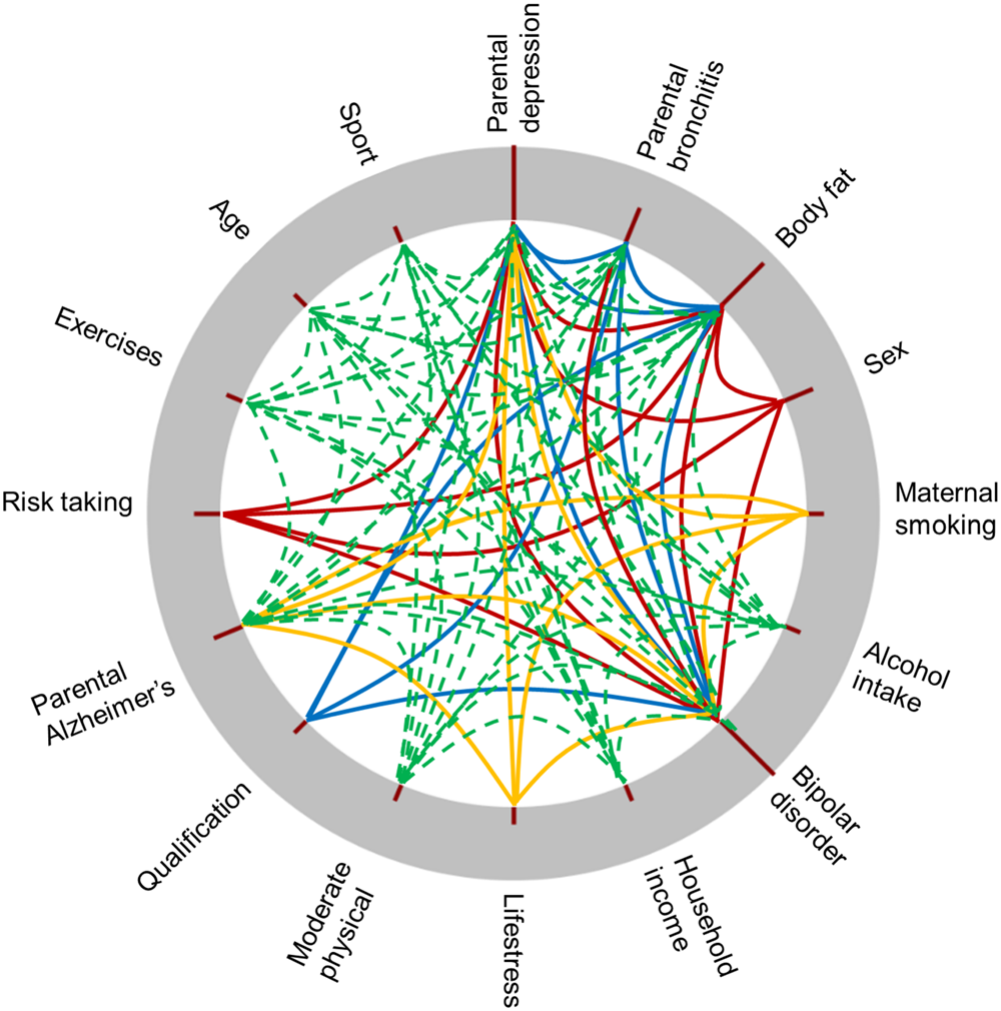
Environment-environment interactions. Variables connected with the same marker represent strongly relevant sets of variables that form higher-order interactions affecting lifetime depression. The height of the red column for a given variable corresponds to the probability of relevance of that variable.

Figure 2 shows possible structural interactions among elements of each relevant variable set using differently colored markers. Variables representing the neuroticism trait, current depressive symptoms, and parental depression are present in all sets as direct relationships with individual main effects. In addition, parental depression plays a central role in several structural interactions by providing context for interaction terms (this assessment requires the analysis of possible dependency structures of variables not discussed here in details). Similarly, body fat percentage (*p*_*Dir*_ = 0.800), life stress (*p*_*Dir*_ = 0.200) and household income (*p*_*Dir*_ = 0.200) are in a direct relationship with lifetime depression and also play roles in interaction patterns. Bipolar disorder on the other hand is present in almost all relevant sets, but only as an interaction term (*p*_*Int*_ = 0.999).

According to Table 3 the most probable relevant set consists of risk taking, sex, parental depression, body fat percentage, bipolar disorder, current depressive symptoms and neuroticism. Based on Table 2, variables such as risk taking (*p*_*Int*_ = 0.328), sex (*p*_*Int*_ = 0.395) and bipolar disorder (*p*_*Int*_ = 0.999) are potential interaction terms, whereas parental depression, body fat percentage, neuroticism and current depressive symptoms are potential main effects of these structural interactions. The second sets includes qualification (*p*_*Int*_ = 0.200), parental bronchitis (*p*_*Int*_ = 0.477), and bipolar disorder as potential interaction terms. The third set contains several interaction terms out of which exercises (*p*_*Int*_ = 0.200), sports (*p*_*Int*_ = 0.200), moderate physical activity (*p*_*Int*_ = 0.200), age (*p*_*Int*_ = 0.200), alcohol intake (*p*_*Int*_ = 0.200) are more likely to form an interaction pattern with household income and body fat as a main effect (according to additional analysis of possible dependency structures). Finally, the fourth set consists of maternal smoking (*p*_*Int*_ = 0.201), parental Alzheimer’s (*p*_*Int*_ = 0.400), and bipolar disorder as interaction terms and life stress as a main effect instead of body fat percentage.

These results (and structural interactions in general) indicate that based on the dependency structure these variables have a multivariate effect on the target. In most cases the individual relevance of these variables is moderate or low with respect to the target, but they have a higher relevance as a pattern. Although structural interactions do not provide information on the parametric nature of these effects, they can be utilized to direct effect size analysis efforts.

#### Parametric interactions

In most cases when interactions are considered, the parametric aspect of relevance is investigated by applying various effect size measures such as the odds ratio for a binary target. The challenge is that the individual effect of interaction terms tends to be moderate or small, whereas their joint effect is considerably larger. The latter requires a multivariate effect size measure that is capable of computing an odds ratio for value configurations of multiple variables. Since selecting the base configuration of values - against which all other configurations are compared - is non-trivial, we utilized a value configuration relative odds ratio, i.e. a given value configuration is compared against all other possible configurations (see the Multivariate effect size measure section of methods for details).

**Figure 3:**
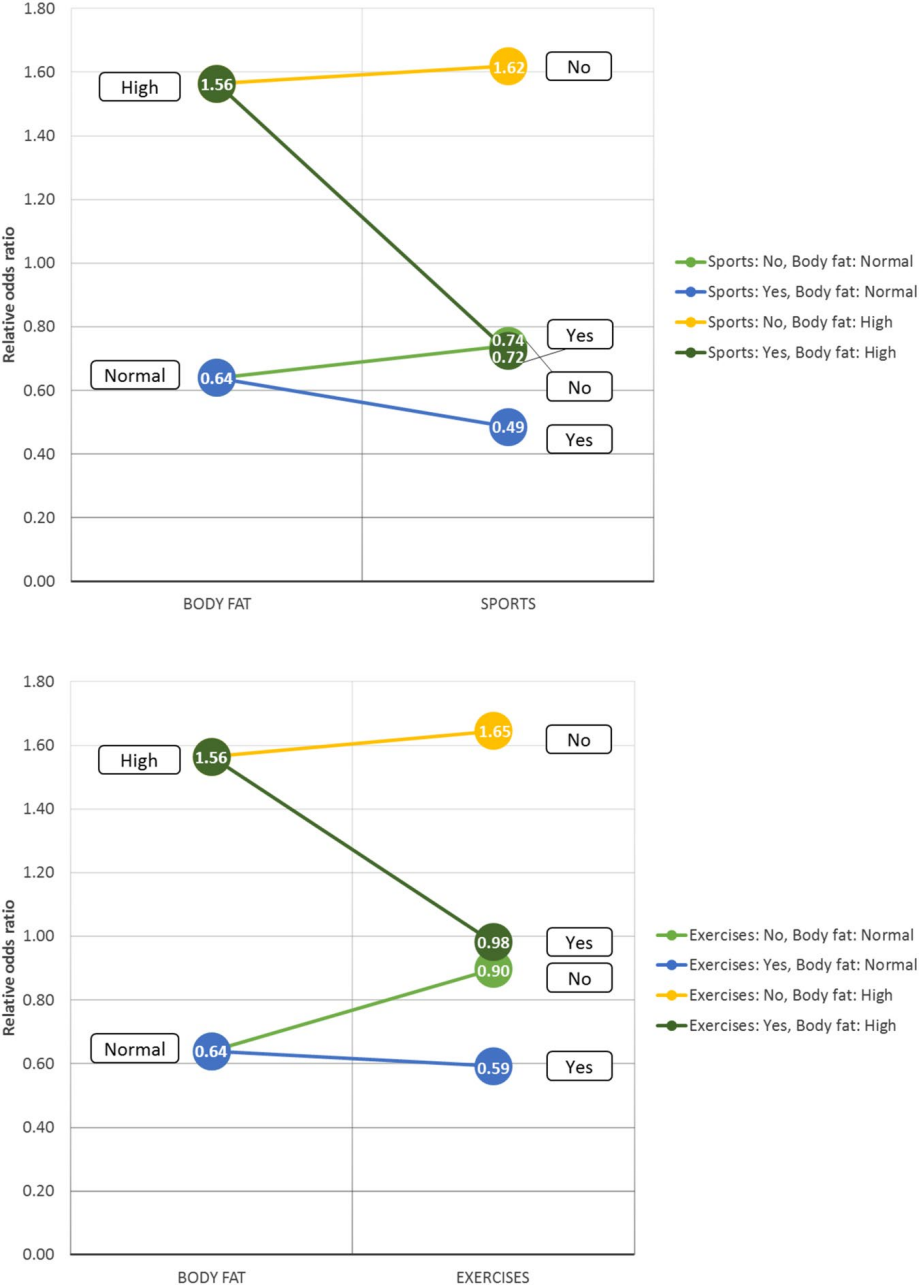
Parametric interactions of Body fat and sporting activity descriptors: Exercises and Sports.

Structural interaction results indicate higher-order interactions among members of relevant variable sets, however subsets of these variables can also be of interest. Furthermore, note that these interactions are interpreted on the level of variables and provide no further insight neither on the value level nor on the parametric level, i.e. variable value configurations. In order to investigate interactions on a parametric level additional analysis is required which involves the arbitrary selection of a subset of variables from a relevant set. For example, we can investigate parametric aspects of the interaction between body fat and sporting activities with respect to lifetime depression based on a relevant set of variables. Note that this selection is arbitrary as any subset of this relevant set could be technically investigated. Table 4 shows the parametric aspect of this interaction involving strenuous sports and exercises. In general, both physical activity types provide a protective effect with respect to lifetime depression, whereas higher body fat percentage presents a risk (OR(high versus normal) = 1.56). Regarding the joint effects of body fat and physical activity descriptors, the protective effect of doing sports or exercises is larger in case of subjects with high body fat percentage (e.g. CR-OR(Sports: No, Body fat: High) = 1.62, CR-OR(Sports: Yes, Body fat: High) = 0.72) than in case of subjects with normal body fat percentage (e.g. CR-OR(Sports: No, Body fat: Normal) = 0.74, CR-OR(Sports: Yes, Body fat: Normal) = 0.49). Compared to strenuous sports, the protective effect of exercises is smaller in both the normal (CR-OR(Exercises: No, Body fat: Normal) = 0.9, CR-OR(Exercises: Yes, Body fat: Normal) = 0.59) and high (CR-OR(Exercises: No, Body fat: High) = 1.56, CR-OR(Exercises: Yes, Body fat: High) = 0.92) body fat subgroups (shown in Fig. 3).

**Table 4:** Parametric interactions of Body fat, Exercises and Sports with respect to lifetime depression.

Body fat (I.)		I. + Sports (II.A)				I. + Exercises (II.B)			
Normal	CR-OR		CR-OR	CI_95%_			CR-OR	CI_95%_	
				Low	High			Low	High
	0.64	No	0.74	0.70	0.78	No	0.90	0.84	0.96
		Yes	0.49	0.43	0.55	Yes	0.59	0.55	0.63
High	1.56	No	1.62	1.53	1.71	No	1.65	1.56	1.74
		Yes	0.72	0.62	0.84	Yes	0.98	0.92	1.04

CR-OR and CI_95%_ denotes the configuration relative odds ratio and its 95% confidence interval respectively.

Additionally, a detailed analysis of several interaction patterns is provided as additional information.

### Synergistic effects

Interactions and synergistic effects can be distinguished based on the constraints they impose on the dependency structure. Whereas interactions can be related to specific dependency structures, synergistic effects are more general in the sense that there is no such hierarchy implied concerning the dependency structure of variables. Although it is reasonable to assume that variables with distinct individual main effects are among the first to be investigated for additional synergistic effects.

**Figure 4:**
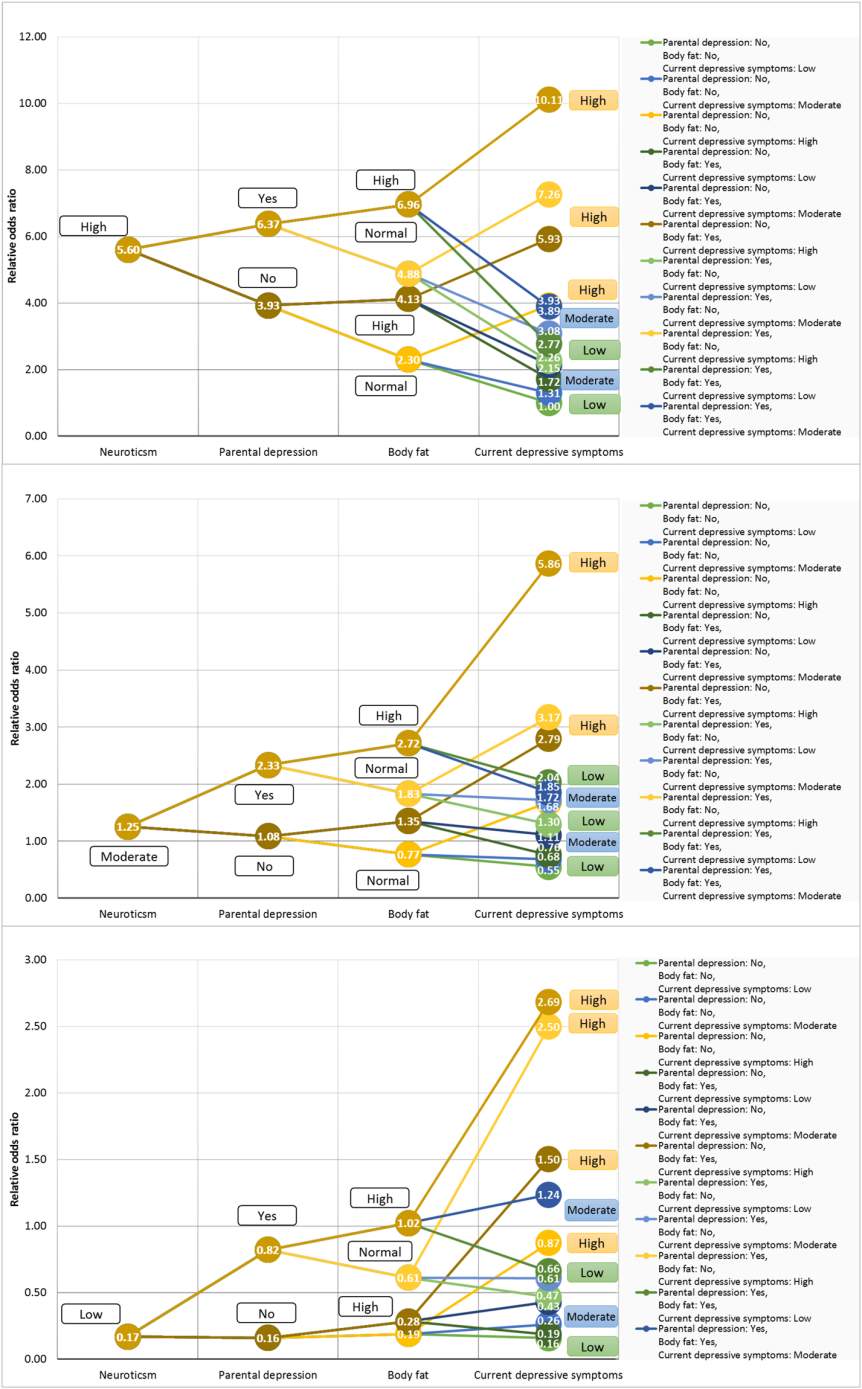
Synergistic effects of Neuroticism, Parental depression, Body fat, and Current depressive symptoms.

In our case Neuroticism, Parental depression, Current depressive symptoms, and Body fat variables can be considered as a set of relevant variables with synergistic effects. The BMLA method identified each of these variables as strongly relevant and directly connected to lifetime depression. As a set of relevant variables, they are part of the majority of possible strongly relevant variable sets. To quantify synergistic effects from a parametric aspect, a multivariate effect size analysis can be performed similarly to that which was applied in case of parametric interactions. Table 5 shows configuration relative odds ratios for various Neuroticism - Parental depression - Body fat - Current depressive symptoms subgroups. Both the neuroticism score and current depressive symptoms affect lifetime depression according to severity, a higher value conveys a larger risk of lifetime depression than a lower one (specifically OR(Neuroticism: Moderate vs. Low) = 3.44, OR(Neuroticism: High vs. Low) = 9.26, OR(Current depressive symptoms: Moderate vs. Low) = 2.41, OR(Current depressive symptoms: High vs. Low) = 10.66). Comparatively, the individual effect of parental depression (OR(Yes vs. No) = 2.84) and body fat (OR(High vs. Normal) = 1.56) is lower. Results in Fig. 4 are displayed separately based on neuroticism categories.

**Table 5:** Synergistic effects of Neuroticism, Parental depression, Body fat, and Current depressive symptoms with respect to reported lifetime depression.

I.		I. + II.		I-II. + III.		I-III. + IV.			
Neuroticism		Parental depression		Body fat		Current depressive symptoms			
	CR-OR		CR-OR		CR-OR		CR-OR	CI_95%_	
								Low	High
Low	0.17	No	0.16	Normal		Low	0.16	0.14	0.19
						Moderate	0.26	0.23	0.31
					0.19	High	0.87	0.63	1.21
				High		Low	0.19	0.16	0.21
						Moderate	0.43	0.38	0.48
					0.28	High	1.50	1.22	1.85
		Yes	0.82	Normal		Low	0.47	0.32	0.67
						Moderate	0.61	0.43	0.86
					0.61	High	2.50	1.40	4.47
				High		Low	0.66	0.49	0.89
						Moderate	1.24	0.98	1.57
					1.02	High	2.69	1.66	4.35
Moderate	1.25	No	1.08	Normal		Low	0.55	0.44	0.68
						Moderate	0.68	0.59	0.78
					0.77	High	1.68	1.39	2.03
				High		Low	0.76	0.63	0.91
						Moderate	1.11	1.00	1.23
					1.35	High	2.79	2.47	3.16
		Yes	2.33	Normal		Low	1.30	0.82	2.04
						Moderate	1.72	1.32	2.23
					1.83	High	3.17	2.12	4.74
				High		Low	2.04	1.37	3.03
						Moderate	1.85	1.46	2.35
					2.72	High	5.86	4.54	7.57
High	5.60	No	3.93	Normal		Low	1.00	0.75	1.34
						Moderate	1.31	1.14	1.49
					2.30	High	3.93	3.55	4.34
				High		Low	1.72	1.38	2.15
						Moderate	2.15	1.94	2.38
					4.13	High	5.93	5.49	6.4
		Yes	6.37	Normal		Low	2.26	1.30	3.94
						Moderate	3.08	2.46	3.86
					4.88	High	7.26	6.12	8.62
				High		Low	2.77	1.71	4.50
						Moderate	3.89	3.19	4.74
					6.96	High	10.11	8.84	11.57

In general, the presence of parental depression entails higher risk for lifetime depression, as does high body fat percentage. These effects are more pronounced in subgroups with high neuroticism scores. In addition, the current depressive symptoms variable influences the effect size of a particular configuration to the largest extent. The before-mentioned effects of risk factors are observable especially in case of high current depressive symptom scores. The two extreme points of variable configurations in terms of effect size are: (1) subjects with high neuroticism score, high current depressive symptoms score, high body fat percentage, and with parental depression (CR-OR: 10.11), and (2) subjects with low neuroticism score, low current depressive symptoms score, normal body fat percentage, and no parental depression (CR-OR: 0.16). In terms of ordinary odds ratio, this means that subjects with the former traits are 52.61 times more likely to suffer from depression than subjects with the latter traits.

**Figure 5:**
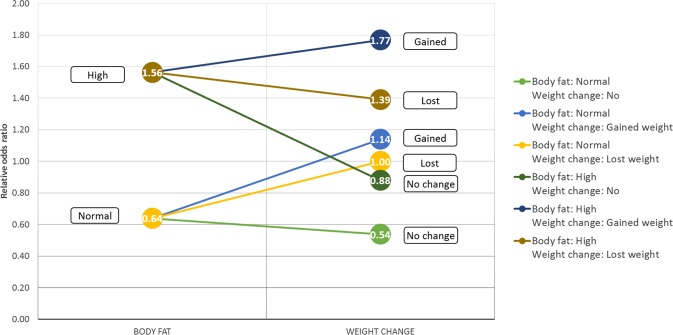
Synergistic effects of Body fat and Weight change.

Furthermore, we investigated another synergistic effect related to body fat percentage which involves weight change and dietary change. Weight change was found transitively relevant with respect to lifetime depression and has a considerable effect size related to depression (CR-OR(Weight change: No change) = 0.57, CR-OR(Weight change: Weight gain) = 1.68, CR-OR(Weight change: Weight loss) = 1.24). Table 6 displays joint effect sizes regarding body fat percentage and weight change. Results indicate that subjects with high body fat percentage have higher risk of lifetime depression compared to similar configurations with normal body fat percentage (see Fig. 5). Concerning weight change, gaining weight conveys a larger risk with respect to lifetime depression than losing weight in both body fat subgroups (e.g. CR-OR(Weight change: Gain, Body fat: High) = 1.77, CR-OR(Weight change: Lost, Body fat: High) = 1.39).

**Table 6:** Synergistic effects of Body fat and Weight change with respect to lifetime depression.

Body fat (I.)		I. + Weight change (II.)			
	CR-OR		CR-OR	CI_95%_	
				Low	High
Normal	0.64	No	0.54	0.50	0.57
		Gained weight	1.14	1.04	1.26
		Lost weight	1.00	0.90	1.11
High	1.56	No	0.88	0.83	0.94
		Gained weight	1.77	1.67	1.88
		Lost weight	1.39	1.28	1.51

CR-OR and CI_95%_ denotes the configuration relative odds ratio and its 95% confidence interval respectively.

Moreover, the potential effect of dietary change was also investigated in conjunction with the effects of body fat and weight change. Supplementary Table S20 shows multivariate effect sizes for various Body fat, Dietary change and Weight change variable configurations. The previously observed effect that weight gain serves as the largest risk factor remains valid in all body fat - dietary change subpopulations.

### Predictive power

In addition to relevance, the predictive power of variables can also be of interest when building a predictive model. In most cases variables that are directly relevant to a target are among those that have the highest predictive power with respect to the target. However, it should be also taken into account that (1) some strongly relevant variables are highly predictive only in a multivariate context, i.e. as part of a set of predictor variables, and (2) there are variables that are only transitively relevant but are highly predictive.

In order to investigate the predictive nature of specific variable sets and also individual variables we utilized a deep neural network based classifier having lifetime depression as the target (class) variable. For the purposes of evaluation we used an information theoretic measure (cross-entropy), residual variance, and a predictive performance measure. The reduction of residual variance due to a feature was also computed by comparing the measured residual variance to that of a random classifier. Furthermore, the predictive performance of a feature was also compared to the performance of a saturated model containing all variables (for further details see the Deep neural network based modeling section in methods).

Table 7 displays measures related to some of the most significant variables in terms of association with lifetime depression. Results indicate that the neuroticism trait and current depressive symptoms are the most relevant variables as they have the best scores both in terms of reduction of residual variance (19.02% and 16.67% respectively) and of predictive performance (97.17% and 88.05% relative to the saturated model). Other variables achieve remarkably lower reduction in residual variance. Parental depression is also among the highly predictive variables (predictive rank: 3). Interestingly, the variable describing satisfaction (association rank: 3) has one of the lowest scores among these variables in terms of residual relevance reduction (1.21%, rank: 10) and also in terms of predictive performance (75.4%, rank: 11). Life stress and household income have smaller predictive power (79.41%, rank: 4 and 78.02%, rank: 7 respectively) compared to parental depression. Similarly, the BMLA method detected both variables having a direct relationship with lifetime depression but with a considerably lower probability than parental depression. To the contrary, Headache was not detected as a relevant variable even though it achieved better results in residual variance reduction (5.46%, rank: 3) than life stress (2.93%, rank: 7) and slightly better predictive power (78.53%, predictive rank: 6) than household income (78.02%, predictive rank: 7). According to the envirome map, this lack of a direct effect of headache on lifetime depression is probably due to the mediatory role of neuroticism and current depressive symptoms. In other words, despite the fact that the presence of headache has a highly significant association with lifetime depression (association rank: 5) and has considerable predictive power, these properties do not entail strong relevance.

**Table 7:** Predictive measures of variables having a highly significant association with lifetime depression.

Variable	Association		Residual variance			Predictive performance		
	−Log (p)	Rank	Variance	Reduction	Rank	Score	Ratio	Rank
Neuroticism	800	1	0.203	19.02%	1	0.702	97.17%	1
Current depressive symptoms	800	2	0.208	16.67%	2	0.636	88.05%	2
Satisfaction	703.3	3	0.247	1.21%	10	0.545	75.40%	11
Parental depression	528.1	4	0.238	4.93%	4	0.626	86.61%	3
Headache	388.8	5	0.236	5.46%	3	0.568	78.53%	6
Life stress	377.3	6	0.243	2.93%	7	0.574	79.41%	4
Falls	344.3	7	0.243	2.99%	6	0.560	77.44%	8
Household	291.2	8	0.246	1.47%	9	0.564	78.02%	7
Insomnia	283.7	9	0.242	3.04%	5	0.550	76.15%	10
Weight change	237.4	10	0.249	0.30%	11	0.570	78.85%	5
Body fat	129.9	11	0.246	1.52%	8	0.556	76.89%	9

*−Log (p)* denotes the negative logarithm of the p-value related to the association test. *Reduction* denotes the residual variance reduction compared to the random classifier. *Ratio* stands for the ratio of the predictive performance score and to that of a saturated model.

Furthermore, the body fat descriptor is among the less predictive variables (individually) on this list (predictive rank: 9). In a multivariate context however, body fat along with neuroticism, current depressive symptoms and parental depression forms one of the most highly predictive variable sets with respect to lifetime depression. Supplementary Table S19 shows the predictive performance of this set and that of the most relevant sets of variables identified by the BMLA.

In addition, the predictive power of variable groups (e.g. social factors, financial background, etc.) was also investigated using a deep neural network classifier. In these cases only the variables related to a selected group formed the input layer of the network. Table 8 displays cross-entropy, residual variance and predictive performance measures and corresponding ranks for each variable group. Cross-entropy measured the remaining uncertainty between predicted and actual outputs of the classifier. According to results, mental health descriptors have the highest predictive power with respect to lifetime depression, followed by pain descriptors, diet and metabolism factors, life stress and parental illnesses. Note that childhood descriptors only consisted of general factors such as body size, height, and maternal smoking. Childhood trauma and maltreatment indicators (with the exception of first sexual intercourse) were not included in this analysis.

**Table 8:** The predictive power of variable groups with respect to lifetime depression.

Variable group	Cross-entropy		Residual variance			Predictive performance		
	Score	Rank	Score	Reduction	Rank	Score	Ratio	Rank
Mental health	2.591	1	0.191	23.71%	1	0.713	98.67%	1
Pain	2.875	2	0.235	6.08%	2	0.594	82.23%	2
Parental illnesses	2.899	3	0.241	3.75%	3	0.570	78.85%	5
Diet and metabolism	2.900	4	0.243	2.91%	7	0.582	80.51%	3
Life stress	2.908	5	0.242	3.30%	5	0.574	79.37%	4
Financial background and qualification	2.908	6	0.241	3.67%	4	0.564	78.08%	7
Sports and physical activity	2.914	7	0.243	2.86%	8	0.560	77.43%	10
Age and sex	2.917	8	0.246	1.72%	11	0.565	78.23%	6
Falls	2.917	9	0.243	2.99%	6	0.560	77.44%	9
Tobacco and alcohol consumption	2.919	10	0.246	1.76%	10	0.565	78.13%	8
Social factors	2.928	11	0.245	1.99%	9	0.549	75.95%	11
Blood pressure	2.930	12	0.247	1.17%	12	0.544	75.25%	12
Childhood descriptors	2.938	13	0.250	0.15%	13	0.526	72.81%	13

*Reduction* denotes the residual variance reduction compared to the random classifier. *Ratio* stands for the ratio of the predictive performance score and to that of a saturated model.

The superior predictive performance of the mental health group (0.713) was expected as it contained two of the most relevant variables (neuroticism and current depressive symptoms) which also had the highest predictive power. In addition, the sleep quality descriptor (Insomnia) also contributed to the predictive power.

Pain descriptors appear to be the second most predictive group (0.594) containing all pain related variables such as headache. This result indicates that various types of pain descriptors can be correlated with lifetime depression, however in the multivariate context of the environmental factors, these relationships are mediated by other factors.

The third most predictive group is diet and metabolism factors (0.582) which consists of body fat which was found directly relevant with respect to lifetime depression, and several transitively relevant factors such as weight change and the obesity descriptor. In the context of the envirome, this variable group is relevant as some of its effects directly influence lifetime depression.

Following life stress, the parental illnesses variable group is the fifth in the predictive ranking (0.570). Previous analyses revealed that parental depression is the only directly relevant variable within this group, and parental Alzheimer’s disease and bronchitis may play roles in interactions of moderate relevance. As this group represents the hereditary aspect of lifetime depression, these results (i.e. several groups of environmental factors are better predictors) also confirm that investigating environmental factors is essential in predicting depression.

## Discussion

Our study investigating the effects of environmental, social, lifestyle, metabolic and mental health factors on lifetime depression have shown that a surprisingly small number of core factors mediates the effects of the envirome. That is the majority of the envirome variables do not have an independent direct and relevant effect and they are only indirectly related to lifetime depression, exerting their effects in a dynamic network via transitive, interactive and synergistic relationships. This may also explain why existing environmental effects may be obscured in most studies which consider them individually in an isolated way. This result also implies that a narrowly focused set of factors can shield the effects of the whole envirome, suggesting therapeutic, clinical and pharmacological consequences. This drastic reduction of the set of relevant variables related to MDD by filtering the mediated, non-direct factors was also observed in an earlier study, which investigated the ratio of direct multi-morbidities of MDD among all the statistically associated co-morbidities^25^.

In our present study the only directly related and also highly relevant factors with respect to lifetime depression were neuroticism, current depressive symptoms, parental depression, and body fat percentage, which factors also mediated the majority of other effects in various ways. First, current depressive symptoms play a central role mediating the effects of a wide range of variables on lifetime depression including financial factors, sporting activity (partially), social factors (partially), insomnia (partially) and most pain descriptors. Second, neuroticism mediates the effects of social factors (partially), insomnia (partially), and some of the pain descriptors (headache). Third, body fat mediates the effects of most sporting activity and metabolism-related variables. In addition, body fat and parental depression play significant roles in structural interactions involving variables such as life stress, risk taking, maternal smoking, qualification, sport, alcohol consumption and household income. Furthermore, neural network-based analysis of predictive power also indicated that neuroticism, current depressive symptoms and parental depression have the highest predictive capabilities individually, while body fat had a lower predictive power. However, considered jointly as a set these four variables have the highest predictive power and form a core component of the majority of possible strongly relevant sets of variables.

Parametric analysis, besides confirming the relevance of these four variables also revealed synergistic effects between them. While high neuroticism and high current depressive symptoms had the largest individual quantitative effect on lifetime depression (OR: 9.26 and OR: 10.66 respectively), their synergistic effect coupled with presence of parental depression and high body fat had the largest multivariate odds ratio (CR-OR: 10.11) which indicates a 52.61-fold increased risk for lifetime depression compared to those with low neuroticism, low current depressive symptoms, low body fat and no parental depression (CR-OR: 0.16).

The complex pattern of relationships between the identified core factors and their role in communicating the relationship of several other factors on lifetime depression is novel compared to previous studies^14,15^. These core variables, however, have been previously implicated in association with lifetime depression in individual studies. Nonetheless, a closer look may also reveal their role in influencing the effect of other environmental variables as well. The strong relevance of parental depression with respect to lifetime depression as well as its high predictive capacity found in our study may in part reflect the significant heritability and familial aggregation of major depression^27^. However, while heritability of major depression is estimated from approximately 37% in general population samples^27^ up to 75% in severely depressed recurrent depressive samples^28^, the effects of parental depression on mood disorders in off-springs go beyond genetic transmission^29^. This may determine early environmental influences including rearing and financial conditions, as well as possible early neglect and abuse on the one hand, and transmission of coping strategies and shaping character traits and behaviors by model learning on the other^30–32^. These factors may impact eliciting and responding to depression-relevant environmental events and stressors lasting into adulthood^33^, and may also influence future social, lifestyle and metabolic status^34,35^, although it must be noted that we had not included information on the timing of occurrence of parental depression within the lifetime of our subjects and whether it was paternal or maternal, and our data does not permit drawing conclusions concerning the direction of effects.

Similarly, neuroticism, besides its impact as a vulnerability factor for risk of depression^36–43^, with possible overlapping genetic susceptibility^4,44–46^ is one of the fundamental traits of personality associated with emotional instability, negativity, increased vigilance and reactibility for negative environmental cues and a tendency for maladaptive reactiveness upon stressors^36^. Our present results confirm not only the fundamental role of Neuroticism in lifetime depression but also its central role in mediating effects of other relevant factors within the envirome.

Current depressive symptoms similarly emerged as showing a direct relationship with high relevance and high predictive capacity with respect to lifetime depression, an illness with a strong tendency to manifest in recurring episodes^47^. Our results indicated that the majority of effects and factors considered in our study were mediated via current depressive symptoms which can, on the one hand, be provoked and precipitated by environmental and lifestyle factors and on the other hand they are likely to determine the perception of social, lifestyle and metabolic factors, activity and functioning as well as perception of and reaction to environmental events and stressors^48–52^. Given the recurrent nature of major depression it is crucial to understand the influence of current depressive status including symptom profile and severity on factors determining lifetime depression as well as its position within the envirome.

Body fat percentage showed high relevance with respect to lifetime depression and mediated the effects of several related variables including stress, alcohol and tobacco consumption, physical activity and income. The association and complex relationship between obesity and depression is well-known^25,53^. While depression and consequential changes in appetite and decreased activity and motivation, as well as lower quality of life and side effects of pharmacotherapy may all be involved in weight changes in both directions but mainly weight gain in depressed patients, obesity may also be involved in the increased risk^54,55^ and development of depression highlighting their reciprocal relationship^56^. Our results showing the core relevance of body fat percentage with respect to lifetime depression may also offer a possible model for understanding the association of depression with several obesity and metabolic-linked disorders such as cardiovascular disorders or type II diabetes which present serious public health challenges. Identifying the relationship between obesity and depression is among the key interests of several research studies^57^. Such analyses are typically based on the body mass index (BMI), and in some cases similar relationships concerning body fat are also explored^58,59^. Results indicated that body fat central distribution measured by waist/hip circumference ratio (WHR) had more significant associations with respect to depression symptoms than BMI. Even though BMI and WHR are closely related, these earlier results suggested that different mechanisms were responsible for the associations with depression symptoms. Our results reflect these observations since body fat was found directly relevant to lifetime depression and interacting with several other factors, whereas obesity was detected as transitively relevant, i.e. its effects on depression are mediated by other variables.

In addition to the above core factors, life stress, which has been explored in relation to depression from several aspects in the past decades^14,15,60^ particularly in the context of gene-environment interactions^8,61,62^ and household income reflecting financial hardship, strongly associated with depression in previous studies^63–68^, were also in a direct relationship with lifetime depression with a low but non-negligible probability. However, analysis of interactions indicated that life stress and household income had considerable effects on lifetime depression in conjunction with other factors with negligible individual effects. Life stress formed an interaction pattern with a considerable effect with lesser investigated factors including maternal smoking and parental Alzheimer’s disease, while household income showed a considerable joint effect in interaction with variables reflecting physical activity and alcohol intake. While considerable research focused on the association with physical activity and alcohol intake in depression^69–71^ trying to decipher their role as causative or consequential factors and their risk and protective effects, our study provides information on their position in the context of an envirome network of several interconnected variables which, on the longer term, could also inform research and clinical work on how they can be exploited in decreasing risk or managing symptoms of depression. All other environmental, social, lifestyle and metabolic factors investigated in our study appeared to have effects only in conjunction with our core factors.

Our results thus clearly indicate that environmental, lifestyle, social and other health-related factors in the envirome of depression act mostly in an indirect way on lifetime depression through a small number of core factors with direct effects and in transitive, interactive and synergistic relationships significantly influencing the effects of other members of the envirome network. Therefore, to understand their impact on depression, or the impact of depression on them, and exploit them for understanding, preventing, screening or treating depression as well as finding their role in restoring well-being, functionality and quality of life in depressed patients they have to be conceived together with their position and complex dynamic relationships in the envirome of depression.

The main challenge of this study is the presence of complex dependency patterns among environmental factors and large effect sizes with respect to lifetime depression. These effects result in strong associations between environmental factors and also between these factors and lifetime depression. However, not all significant associations between environmental factors and depression mean direct influence on depression. The BMLA method^8,72^ is applied in this study to provide the necessary tools to distinguish between such dependency relationship types. In addition, a neural network based classifier was constructed and utilized to enable the assessment of predictive power for selected groups of factors. Our results demonstrated that environment-environment interactions need to be considered when investigating multi-factorial disorders, and multivariate methods are required to explore the relationships involving environmental factors. The presented envirome map of depression is the first comprehensive network of environmental factors that was learned from such a large cohort data. Although it must be noted that the number of included childhood descriptors is limited as childhood trauma related variables were available for only a subset of the investigated cohort and thus were not considered in the analysis.

## Methods

### UK Biobank data set

We analyzed a subset of the UK Biobank data set with a sample size of 110,599 involving subjects that completed the mental health questionnaire (UK Biobank Resource under Application Number 1602). The analyses were focused on lifetime depression (binary, reported by the participants and elaborated by trained research nurses) as the main phenotype descriptor (i.e. target variable). In addition, probable depression diagnosis based on experience of depressed mood and help-seeking for mental health (by Smith *et al*.^26^) was utilized as a secondary target for a comparative analysis. Investigated variables (57) include: (A) Mental health descriptors, (B) Social factors, (C) Childhood descriptors, (D) Parental illnesses, (E) Lifestyle - sports, (F) Alcohol and tobacco consumption, (G) Diet and metabolism (including blood pressure and pulse rate), (H) Financial background and qualification, (I) Pain, (J) Life stress, (K) Falls, (L) Age, sex and handedness.

Since the applied BMLA method only allows categorical variables to be analyzed, categorization was performed when needed on continuous variables. Supplementary Tables S1–S4 display the distribution of possible values for each categorical variable included in the analysis. In addition, Supplementary Tables S5–S16, describe the derivation protocol for each variable, and Supplementary Tables S17 and S18 show their individual effect size with respect to lifetime depression.

Categorization levels were based on standard thresholds when they were available, e.g. in case of systolic and diastolic blood pressure. In case of complex variables which aggregate several items, such as current depressive symptoms and neuroticism, weighted summary scores were computed and then categorized based on experts’ advice. In cases where no guidelines were available, the variable levels were selected based on the distribution of values. Note that given the reasonably large sample size available in this study, we aimed to have a detailed categorization of variables where applicable, i.e. we defined multiple categories instead of having dichotomous variables. Since categorization levels were selected to correspond to practically available and semantically appropriate categories, this approach ensures that the number of levels does not contradict some essential property of the variable, and provide robust results.

The need for categorizing variables can be considered as a limitation of this study and its results. However, the main conclusions of the study should not change even if the number of levels were changed within a reasonable interval. In addition, post-hoc effect size analyses have shown that the categorization level of variables can be considered appropriate because the direction of observed effects (based on these categories) reflect previously published results.

### Validation of the investigated depression phenotype

In addition to lifetime depression, we investigated relevant relationships with respect to probable depression diagnosis variables: single depressive episode, moderate depression, and severe depression created by Smith *et al*.^26^. We performed a comparative analysis to identify differences among relevant factors related to various phenotypes. Table 9 displays relevant factors with respect to lifetime depression and probable depression diagnosis variables treated as a group, i.e a single posterior probability of relevance is calculated for each variable with respect to the targets.

**Table 9:** Relevance of variables with respect to reported lifetime depression and to probable depression diagnosis.

Variable	Lifetime depression	Depression (Smith *et al*.)
Sex	0.395	1.000
Age	0.200	0.250
Current depressive symptoms	1.000	1.000
Parental depression	1.000	1.000
Bipolar disorder	0.999	1.000
Neuroticism	0.999	1.000
Risk taking	0.328	0.253
Life stress	0.200	0.251
Satisfaction	0.000	0.500
Body fat	0.800	0.250
Dietary change	0.000	0.496
Obesity	0.000	0.250
Parental bronchitis	0.477	0.500
Parental Alzheimer’s	0.400	0.001
Maternal smoking	0.201	0.001
Moderate physical activity	0.200	0.001
Exercises (pleasure)	0.200	0.251
Sports (strenuous)	0.200	0.251
Qualification	0.200	0.002
Household income	0.200	0.251
Alcohol intake	0.200	0.500

Results indicate that current depressive symptoms, neuroticism, and parental depression are highly relevant in both cases. Furthermore, several moderately relevant factors such as risk taking, life stress, age, household income, exercises, and sports have similar probabilities of relevance. A notable difference between the relevant factors of lifetime depression and probable depression diagnosis is that metabolism related variables act differently. In case of lifetime depression only body fat percentage is relevant with a high posterior probability (*p*_*Rel*_ = 0.8), whereas in case of probable depression diagnosis that relevance is ‘distributed’ between body fat percentage (*p*_*Rel*_ = 0.25), obesity (*p*_*Rel*_ = 0.25), and the dietary change variable (*p*_*Rel*_ = 0.496). Maternal smoking, moderate physical activity and qualification appear to be non-relevant (*p*_*Rel*_ < 0.001) with respect to probable depression diagnosis, compared to the low but non-negligible relevance with respect to lifetime depression (*p*_*Rel*_ = 0.2). On the other hand, variables such as sex, the level of satisfaction and alcohol intake are more relevant with respect to probable depression diagnosis.

All in all, lifetime depression and probable depression diagnosis appear to be adequately similar in terms of relevant factors considering that the frequency of depression cases is 5.2% for lifetime depression and 18.5% in case of probable depression diagnosis.

### A Bayesian framework for identifying dependency relationships

We applied Bayesian networks in the Bayesian statistical framework to explore dependency, causal and relevance relationships between variables based on data. A Bayesian network is a probabilistic graphical model *BN*(*G*,*θ*), in which a *directed acyclic graph* (DAG) *G* represents the multivariate dependency relationships of variables, and parametrization *θ* quantitatively defines the dependency relationships by conditional probability distributions. We applied a DAG-based Markov Chain Monte Carlo (MCMC) method referred to as the Bayesian multilevel analysis of relevance (BMLA) to infer posteriors of complex structural patterns, which represent systematic, scalable repertoires of dependency, causal and relevance relationships^24,73^. In the following sections we introduce some of the corresponding fundamental concepts.

#### Strong relevance

Structural properties of Bayesian networks express various forms of structural relevance. An essential relevance measure called strong relevance is related to the concept of minimal Markov blanket (a.k.a. Markov Boundary Set)^74^. Assuming that a given graph *G* properly represents all dependency relationships defined by the data, the Markov blanket set is represented by the neighborhood of a node (variable) *Y* which consists of those variables *X*_*i*_ that are in either direct structural relationship with *Y* or that are interaction terms. In other words, the Markov blanket set of *Y* ‘isolates’ *Y* from the effects of the rest of the network, such that if the values of the variables within the Markov blanket are known then no further information is required to infer the value of *Y*. Throughout the paper we refer to the Markov blanket set as strongly relevant set of variables which are represented by structural properties (i.e. nodes) of a Bayesian network. In the Bayesian framework a posterior probability can be induced for several types of structural properties. The posterior of strong relevance of a variable *X*_*i*_ can be estimated by model averaging, that is assessing the probability mass of those Markov blanket sets of which *X*_*i*_ is a member. The higher is the sum of posterior probabilities related to Markov blanket sets of which *X*_*i*_ is part of, the more relevant *X*_*i*_ is considered.

Technically, for each variable *X*_*i*_ an indicator function called Markov blanket membership can be defined as ${I}_{MBM({X}_{i},Y,G)}$, which takes the value of 1 if *X*_*i*_ is a member of the Markov blanket of *Y* in DAG structure *G*. Assuming that the data *D* admits multiple possible dependency structures (i.e. models) *G*, and that the indicator function can be evaluated for each such *G*, then the posterior probability of strong relevance *P*(*MBM*(*X*_*i*_, *Y*, *G*|*D*)) can be defined by model averaging^75^ based on the posteriors of possible structures^21,76^. Therefore, strong relevance can be considered as an aggregate of possible multivariate dependency models in the form of a pairwise relevance measure between *X*_*i*_ and *Y*. The challenge of this approach is to properly assess the posterior probability of structural features such as Markov blanket sets.

#### Bayesian statistical framework

Relying on model averaging, the general task would be to compute the posterior of selected structural properties over the space of possible DAG structures G. However, due to the high cardinality of possible DAG structures, the exact computation of such posteriors is generally infeasible. Instead, various approximation methods using a Markov Chain Monte Carlo (MCMC) scheme over the space of DAGs were developed^22,23^ and improved^77,78^.

In order to efficiently estimate the posteriors of complex structural patterns representing potential dependency, causal and relevance relations, especially the complex patterns of higher-order interactions, we introduced subtypes of relevances to bridge the gap between the pairwise Markov Blanket Membership and multivariate Markov Blanket Set^24^. The methodology and corresponding toolset is referred to as the Bayesian multilevel analysis of relevance (BMLA)^24^. The BMLA method can perform MCMC over the space of DAGs, PDAGs and orderings. In the current paper we applied the method in the space of DAGs using the unnormalized posterior to aid the random walk. The alteration between various DAG structures (i.e. transition between states) is facilitated by special operators deleting, inverting or inserting edges^77^.

In an optimal case, the MCMC process reaches a stationary state after a number of steps. The collected samples are utilized for the computation of posteriors only after the burn-in period, which is (approximately) the interval preceding convergence. The result of this process is a set of DAG structures which can be used to evaluate various structural properties such as direct edges or more complex properties such as Markov blanket sets. In order to compute strong relevance with respect to a target *Y*, the Markov blanket set of *Y* is identified in each sampled *G* structure and subsequently Markov blanket membership functions are evaluated (${I}_{MBM({X}_{i},Y,G)}$). By applying model averaging, the latter can be used to estimate the posterior probability of strong relevance for each *X*_*i*_ with respect to a selected target *Y*. This means that each instantiation of the investigated structural property is registered and corresponding frequency information is collected, and as a last step a normalization is performed to ensure that the results can be interpreted as probabilities [0, 1].

Note that BMLA can handle the redundancy of variables automatically. Redundancy from the perspective of Markov blanket sets means that e.g. variable *X*_*k*_ is not present in a Markov blanket set of target *Y* if variable *X*_*i*_ is already a member of that set. If however *X*_*i*_ is not present in a Markov blanket set of *Y* then *X*_*k*_ can be a member (assuming that it is relevant to some extent with respect to *Y*). If variable *X*_*k*_ is redundant (with respect to *Y*) then that is reflected in a relatively lower relevance score since it is present in a smaller number of possible models.

#### Deep neural network based classifier

In order to measure the predictive power of environmental factors, and also relevant sets of environmental factors, we utilized a deep neural network^79,80^ based classifier using the Tensorflow framework^81^. The network consisted of three fully connected layers using ReLU (rectified linear unit) activation functions, containing neurons matching the number of variables in the study, and an output layer. Weights and biases were initialized randomly according to the uniform distribution. Binary-cross entropy was utilized as loss function, the ADAM method was used as an optimizer^82^.

The evaluation was based on a weighted accuracy measure taking into account the unbalanced case-control ratio (i.e. subjects with and without depression). In addition, residual variance and cross-entropy was computed between the actual and predicted values. Residual variance values were compared to the residual variance of a random classifier thereby measuring the reduction in its value. Weighted accuracy measures were compared to the performance of a saturated model including all variables. All measures were computed using a *k* = 10 fold cross-validation, i.e. the data was split into k = 10 partitions and for each phase 9 partitions were used as training data and 1 partition as testing data. This process was repeated n = 10 times.

#### Multivariate effect size measure

Effect size measures are quantitative and are typically utilized in a pairwise form describing the effect of one discrete variable on another. For example an odds ratio of a variable *X* with respect to a target trait *Y* defines the extent that a change in the value of *X* causes in the conditional distribution of *Y*. By convention if the target is binary then odds are computed such that the numerator is related to the cases, and the denominator is related to controls, otherwise a base value is selected. Straightforwardly, odds ratios are computed using odds corresponding to various values of *X* such that one of the values is selected to be the base. If there are multiple values of *X* then multiple odds ratios can be defined according to the selected base. The challenge arises when the selection of a base value is non-trivial, because for example *X* has six possible values (*x*_1_, *x*_2_, …, *x*_6_) and neither the smallest nor the largest value is a good choice for a base value. Then a possible solution is to apply value relative odds ratio which means that each odds related to a certain value of *X* (e.g. *X* = *x*_1_) is compared against the odds related to all other values (e.g. *X* = *x*_2_, *x*_3_, …, *x*_6_): Odds(*X* = *x*_1_)/Odds(*X* ≠ *x*_1_). In this case no base value selection is required, and the effect size is more robust as more samples are utilized.

In a multivariate case, the aim is to measure the joint effect of two or more variables (e.g. *X* and *Z*) on a single target *Y*. Considering odds ratios with a binary target, this means that for every configuration of *X* and *Z* an odds has to be computed. However, depending on the number and cardinality of involved variables, the number of configurations can be considerably large. Therefore, defining an appropriate base value for odds ratios can be even more challenging than in the pairwise case. Instead, utilizing the previously presented idea, a configuration relative odds ratio (CR-OR) can be defined such that an odds for a given configuration of values (e.g. Odds(*X* = *x*_1_, *Z* = *z*_1_)) is compared to an odds corresponding to all other possible configurations Odds(*X* ≠ *x*_1_, *Z* ≠ *z*_1_), that is CR-OR(*X* = *x*_1_, *Z* = *z*_1_) = Odds(*X* = *x*_1_, *Z* = *z*_1_)/Odds(*X* ≠ *x*_1_, *Z* ≠ *z*_1_). This multivariate effect size measure provides a robust view on the joint effect of multiple examined variables on a single target.

## Supplementary information

Supplementary Information

## Data Availability

This study only utilized the UK Biobank dataset (application number 1602) under standard terms and conditions.

## References

[1] Wittchen HU (2011). The size and burden of mental disorders and other disorders of the brain in europe 2010. Eur. neuropsychopharmacology.

[2] Wittchen, H.U. *The burden of mood disorders* (2012). 10.1126/science.1230817 23042853

[3] Vos T (2016). Global, regional, and national incidence, prevalence, and years lived with disability for 310 diseases and injuries, 1990–2015: a systematic analysis for the global burden of disease study 2015. The Lancet.

[4] Wray NR (2018). Genome-wide association analyses identify 44 risk variants and refine the genetic architecture of major depression. Nat. genetics.

[5] Sullivan PF, Daly MJ, O’donovan M (2012). Genetic architectures of psychiatric disorders: the emerging picture and its implications. Nat. Rev. Genet..

[6] Howard DM (2018). Genome-wide association study of depression phenotypes in uk biobank identifies variants in excitatory synaptic pathways. Nat. communications.

[7] Sham PC, Purcell SM (2014). Statistical power and significance testing in large-scale genetic studies. Nat. Rev. Genet..

[8] Gonda X (2018). Significance of risk polymorphisms for depression depends on stress exposure. Sci. reports.

[9] Kovacs D (2016). Effects of il1b single nucleotide polymorphisms on depressive and anxiety symptoms are determined by severity and type of life stress. Brain, behavior, immunity.

[10] Juhasz G (2014). Brain galanin system genes interact with life stresses in depression-related phenotypes. Proc. Natl. Acad. Sci..

[11] Juhasz G (2011). The creb1-bdnf-ntrk2 pathway in depression: multiple gene-cognition-environment interactions. Biol. psychiatry.

[12] Rivera M (2017). Interaction between the fto gene, body mass index and depression: meta-analysis of 13701 individuals. The Br. J. Psychiatry.

[13] Peyrot W (2015). The association between lower educational attainment and depression owing to shared genetic effects? results in˜ 25 000 subjects. Mol. psychiatry.

[14] Kendler KS, Gardner CO, Prescott CA (2002). Toward a comprehensive developmental model for major depression in women. Am J Psychiatry.

[15] Kendler KS, Gardner CO, Prescott CA (2006). Toward a comprehensive developmental model for major depression in men. Am. J. Psychiatry.

[16] Winham SJ, Biernacka JM (2013). Gene–environment interactions in genome-wide association studies: current approaches and new directions. J. Child Psychol. Psychiatry.

[17] Pearl Judea (1988). MARKOV AND BAYESIAN NETWORKS. Probabilistic Reasoning in Intelligent Systems.

[18] Friedman J, Hastie T, Tibshirani R (2008). Sparse inverse covariance estimation with the graphical lasso. Biostat..

[19] Maathuis MH, Colombo D, Kalisch M, Bühlmann P (2010). Predicting causal effects in large-scale systems from observational data. Nat. Methods.

[20] Nandy P (2017). Estimating the effect of joint interventions from observational data in sparse high-dimensional settings. The Annals Stat..

[21] Cooper GF, Herskovits E (1992). A bayesian method for the induction of probabilistic networks from data. Mach. learning.

[22] Madigan D, Andersson SA, Perlman M, Volinsky CT (1996). Bayesian model averaging and model selection for Markov equivalence classes of acyclic digraphs. Comm.Stat. Theory Methods.

[23] Friedman N, Koller D (2003). Being Bayesian about network structure. Mach. Learn..

[24] Antal, P., Millinghoffer, A., Hullám, G., Szalai, C. & Falus, A. A bayesian view of challenges in feature selection: feature aggregation, multiple targets, redundancy and interaction. In *New Challenges for Feature Selection in Data Mining and Knowledge Discovery*, 74–89 (2008).

[25] Marx P (2017). Comorbidities in the diseasome are more apparent than real: What bayesian filtering reveals about the comorbidities of depression. PLoS computational biology.

[26] Smith DJ (2013). Prevalence and characteristics of probable major depression and bipolar disorder within uk biobank: cross-sectional study of 172,751 participants. PloS one.

[27] Sullivan PF, Neale MC, Kendler KS (2000). Genetic epidemiology of major depression: review and meta-analysis. Am. J. Psychiatry.

[28] Uher R (2014). Gene–environment interactions in severe mental illness. Front. psychiatry.

[29] Bezdjian S, Tuvblad C, Wang P, Raine A, Baker LA (2014). Motor impulsivity during childhood and adolescence: A longitudinal biometric analysis of the go/no-go task in 9-to 18-year-old twins. Dev. psychology.

[30] Natsuaki MN (2014). Raised by depressed parents: is it an environmental risk?. Clin. child family psychology review.

[31] Yap MBH, Pilkington PD, Ryan SM, Jorm AF (2014). Parental factors associated with depression and anxiety in young people: A systematic review and meta-analysis. J. affective disorders.

[32] McAdams T (2015). The relationship between parental depressive symptoms and offspring psychopathology: evidence from a children-of-twins study and an adoption study. Psychol. medicine.

[33] Barry TJ (2015). Maternal postnatal depression predicts altered offspring biological stress reactivity in adulthood. Psychoneuroendocrinology.

[34] Raposa E, Hammen C, Brennan P, Najman J (2014). The long-term effects of maternal depression: early childhood physical health as a pathway to offspring depression. J. Adolesc. Heal..

[35] Plant D, Pawlby S, Sharp D, Zunszain P, Pariante C (2016). Prenatal maternal depression is associated with offspring inflammation at 25 years: a prospective longitudinal cohort study. Transl. psychiatry.

[36] Jeronimus B, Kotov R, Riese H, Ormel J (2016). Neuroticism’s prospective association with mental disorders halves after adjustment for baseline symptoms and psychiatric history, but the adjusted association hardly decays with time: a meta-analysis on 59 longitudinal/prospective studies with 443 313 participants. Psychol. Medicine.

[37] Alnæs R, Torgersen S (1997). Personality and personality disorders predict development and relapses of major depression. Acta Psychiatr. Scand..

[38] Roberts SB, Kendler KS (1999). Neuroticism and self-esteem as indices of the vulnerability to major depression in women. Psychol. medicine.

[39] Kendler KS, Gatz M, Gardner CO, Pedersen NL (2006). Personality and major depression: A swedish longitudinal, population-based twin study. Arch. Gen. Psychiatry.

[40] Pekka J, Tarja M, Heikki R, Erkki I (2009). Neuroticism, introversion, and major depressive disorder—traits, states, or scars?. Depress. Anxiety.

[41] Xia J (2011). The relationship between neuroticism, major depressive disorder and comorbid disorders in chinese women. J. affective disorders.

[42] Juhasz G (2009). Cnr1 gene is associated with high neuroticism and low agreeableness and interacts with recent negative life events to predict current depressive symptoms. Neuropsychopharmacol..

[43] Anttila V (2018). Analysis of shared heritability in common disorders of the brain. Sci..

[44] Fanous A, Gardner C, Prescott C, Cancro R, Kendler K (2002). Neuroticism, major depression and gender: a populationbased twin study. Psychol. Medicine.

[45] Howard, D. M. *et al*. The stratification of major depressive disorder into genetic subgroups. *bioRxiv 134601* (2017).

[46] Hettema JM, Neale MC, Myers JM, Prescott CA, Kendler KS (2006). A population-based twin study of the relationship between neuroticism and internalizing disorders. Am. J. Psychiatry.

[47] Burcusa SL, Iacono WG (2007). Risk for recurrence in depression. Clin. psychology review.

[48] Bourke C, Douglas K, Porter R (2010). Processing of facial emotion expression in major depression: a review. Aust. New Zealand J. Psychiatry.

[49] Leppänen JM (2006). Emotional information processing in mood disorders: a review of behavioral and neuroimaging findings. Curr. opinion psychiatry.

[50] Romera I (2012). Early vs. conventional switching of antidepressants in patients with mdd and moderate to severe pain: a double-blind randomized study. J. affective disorders.

[51] Ushinsky A, Reinhardt LE, Simmons AN, Strigo IA (2013). Further evidence of emotional allodynia in unmedicated young adults with major depressive disorder. PloS one.

[52] Weightman MJ, Air TM, Baune BT (2014). A review of the role of social cognition in major depressive disorder. Front. Psychiatry.

[53] Milaneschi, Y., Simmons, W. K., Rossum, E. F. & Penninx, B. W. Depression and obesity: evidence of shared biological mechanisms. *Mol. psychiatry* **1** (2018). 10.1038/s41380-018-0017-5 29453413

[54] Atlantis E, Baker M (2008). Obesity effects on depression: systematic review of epidemiological studies. Int. journal obesity.

[55] Onyike CU, Crum RM, Lee HB, Lyketsos CG, Eaton WW (2003). Is obesity associated with major depression? results from the third national health and nutrition examination survey. Am. journal epidemiology.

[56] Luppino FS (2010). Overweight, obesity, and depression: a systematic review and meta-analysis of longitudinal studies. Arch. general psychiatry.

[57] DeWit LM, Van Straten A, Van Herten M, Penninx BW, Cuijpers P (2009). Depression and body mass index, a u-shaped association. BMC public health.

[58] Rosmond R, Lapidus L, Mårin P, Björntorp P (1996). Mental distress, obesity and body fat distribution in middle-aged men. Obes..

[59] Rosmond R, Björntorp P (1998). Psychiatric ill-health of women and its relationship to obesity and body fat distribution. Obes..

[60] Monroe SM, Reid MW (2009). Life stress and major depression. Curr. Dir. Psychol. Sci..

[61] Caspi A (2003). Influence of life stress on depression: moderation by a polymorphism in the 5-htt gene. Sci..

[62] Culverhouse R (2018). Collaborative meta-analysis finds no evidence of a strong interaction between stress and 5-httlpr genotype contributing to the development of depression. Mol. psychiatry.

[63] Butterworth P, Rodgers B, Windsor TD (2009). Financial hardship, socio-economic position and depression: results from the path through life survey. Soc. science & medicine.

[64] Dunn N (2008). Does perceived financial strain predict depression among young women? longitudinal findings from the southampton women’s survey. Mental health family medicine.

[65] Andrews B, Wilding JM (2004). The relation of depression and anxiety to life-stress and achievement in students. Br. J. Psychol..

[66] Sarginson J (2014). Neuronal nitric oxide synthase (nos1) polymorphisms interact with financial hardship to affect depression risk. Neuropsychopharmacol..

[67] Gonda X (2016). Financial difficulties but not other types of recent negative life events show strong interactions with 5-httlpr genotype in the development of depressive symptoms. Transl. psychiatry.

[68] Gonda, X. *et al*. Genetic variants in major depressive disorder: From pathophysiology to therapy. *Pharmacol. & therapeutics* (2018). 10.1016/j.pharmthera.2018.09.002 30189291

[69] Hartka E (1991). A meta-analysis of depressive symptomatology and alcohol consumption over time. Addict..

[70] Wang J, Patten SB (2001). A prospective study of sex-specific effects of major depression on alcohol consumption. The Can. J. Psychiatry.

[71] Babiss LA, Gangwisch JE (2009). Sports participation as a protective factor against depression and suicidal ideation in adolescents as mediated by self-esteem and social support. J. Dev. & Behav. Pediatr..

[72] Juhasz G (2015). Variability in the effect of 5-httlpr on depression in a large european population: the role of age, symptom profile, type and intensity of life stressors. PLoS One.

[73] Marx P, Millinghoffer A, Juhász G, Antal P (2016). Joint bayesian modelling of internal dependencies and relevant multimorbidities of a heterogeneous disease. Journal Of Machine Learning Research.

[74] Pearl, J. *Causality: Models, Reasoning, and Inference* (Cambridge University Press, 2000).

[75] Hoeting JA, Madigan D, Raftery AE, Volinsky CT (1999). Bayesian model averaging: A tutorial. Stat. Sci..

[76] Buntine, W. L. Theory refinement of Bayesian networks. In *Proc. of the 7th Conf. on Uncertainty in Artificial Intelligence (UAI-1991)*, 52–60 (Morgan Kaufmann, 1991).

[77] Giudici P, Castelo R (2003). Improving Markov Chain Monte Carlo model search for data mining. Mach. Learn..

[78] Niinimaki, T., Parviainen, P. & Koivisto, M. *Partial order mcmc for structure discovery in bayesian networks. In Proc. of the Twenty-Seventh Conf. on Uncertainty in Artificial Intelligence (UAI-11), Barcelona, Spain, July 14-17, 2011*, 557–564 (2011).

[79] Hinton GE, Osindero S, Teh Y-W (2006). A fast learning algorithm for deep belief nets. Neural computation.

[80] Larochelle, H., Erhan, D., Courville, A., Bergstra, J. & Bengio, Y. An empirical evaluation of deep architectures on problems with many factors of variation. In *Proceedings of the 24th international conference on Machine learning*, 473–480 (ACM, 2007).

[81] Abadi M (2016). Tensorflow: A system for large-scale machine learning. OSDI.

[82] Kingma, D. P. & Ba, J. Adam: A method for stochastic optimization. *arXiv preprint arXiv:1412.6980* (2014).

