# Supplementary material for: The UKB envirome of depression: from interactions to synergistic effects
Source: Sci Rep. 2019 Jul 5;9:9723. doi: 10.1038/s41598-019-46001-5 (PMC6611783; doi:10.1038/s41598-019-46001-5)
Supplement: Supplementary file 1 — Supplementary Information [file 41598_2019_46001_MOESM1_ESM.pdf]

Supplementary information

The UKB envirome of depression: from interactions to synergistic effects

Gabor Hullam[1,2], Peter Antal[1], Peter Petschner[2,3], Xenia Gonda[2,4,5], Gyorgy Bagdy[2,3,4], Bill Deakin[6,7], and Gabriella Juhasz[3,6,8]*

[1] Department of Measurement and Information Systems, Budapest University of Technology and Economics, Budapest, H-1117,Hungary

[2] MTA-SE Neuropsychopharmacology and Neurochemistry Research Group, Hungarian Academy of Sciences, Semmelweis University, Budapest, H-1089, Hungary

[3] Department of Pharmacodynamics, Faculty of Pharmacy, Semmelweis University, Budapest, H-1089, Hungary

[4] NAP2-SE New Antidepressant Target Research Group Semmelweis University, Budapest, H-1089, Hungary

[5] Department of Psychiatry and Psychotherapy, Semmelweis University Budapest

[6] Neuroscience and Psychiatry Unit, Division of Neuroscience and Experimental Psychology, University of Manchester and Manchester Academic Health Sciences Centre, Manchester, M13 9PL, UK

[7] Greater Manchester Mental Health NHS Foundation Trust, Prestwich, Manchester, UK

[8] SE-NAP2 Genetic Brain Imaging Migraine Research Group, Semmelweis University, Budapest, H-1089, Hungary

*juhasz.gabriella@pharma.semmelweis-univ.hu

Population descriptors

Group	Variable	Category	Frequency	
**Mental health**	Depression (lifetime)	No	104794	(94.8%)
		Yes	5805	(5.2%)
	Depression (Smith type)	No	90114	(81.5%)
		Yes	20485	(18.5%)
	Bipolar disorder	No	110296	(99.7%)
		Yes	303	(0.3%)
	Current depressive symptoms	No	45194	(40.9%)
		Mild	49795	(45.0%)
		Severe	15610	(14.1%)
	Insomnia	Never	27632	(25.0%)
		Sometimes	52457	(47.4%)
		Usually	30510	(27.6%)
	Neuroticism	Low	65423	(59.2%)
		Medium	24959	(22.6%)
		High	20217	(18.3%)
	Risk taking	No	81169	(73.4%)
		Yes	29430	(26.6%)
	Satisfaction	High	22077	(20.0%)
		Average	70457	(63.7%)
		Low	18065	(16.3%)
**Social factors**	Social activity	No	78048	(70.6%)
		Yes	32551	(29.4%)
	Visits friends/family	No or rarely	25086	(22.7%)
		Weekly	40265	(36.4%)
		Frequently	45248	(40.9%)
	Confide	Never or rarely	13585	(12.3%)
		Sometimes	38437	(34.8%)
		Frequently	58577	(53.0%)
**Childhood factors**	Body size (childhood)	Thin	35653	(32.2%)
		Plump	17924	(16.2%)
		Average	57022	(51.6%)
	Height size (childhood)	Short	22180	(20.1%)
		Tall	30053	(27.2%)
		Average	58366	(52.8%)
	Breastfed	No	29170	(26.4%)
		Yes	81429	(73.6%)
	Maternal smoking	No	77964	(70.5%)
		Yes	32635	(29.5%)
	First intercourse	< 14	1703	(1.5%)
		$14 - 16$	22503	(20.3%)
		> 16	86393	(78.1%)
**Life stress**	Life stress	None	61901	(56.0%)
		1 - event	35086	(31.7%)
		2 or more events	13612	(12.3%)
**Falls**	Falls	None	89480	(80.9%)
		Only 1	14619	(13.2%)
		More than 1	6500	(5.9%)

Supplementary Table S1. **UK Biobank population descriptors I.**

Group	Variable	Category	Frequency	
**Financial background, qualification**	Townsend	Affluent	71776	(64.9%)
		Mean	18227	(16.5%)
		Deprived	20596	(18.6%)
	Qualification (Higher education)	No	49335	(44.6%)
		Yes	61264	(55.4%)
	Household income	Category I. (Lowest)	18770	(17.0%)
		Category II.	27727	(25.1%)
		Category III.	30865	(27.9%)
		Category IV.	25614	(23.2%)
		Category V.	7623	(6.9%)
**Parental illnesses**	Parental depression	No	99879	(90.3%)
		Yes	10720	(9.7%)
	Parental Parkinson	No	106250	(96.1%)
		Yes	4349	(3.9%)
	Parental Alzheimer's	No	93411	(84.5%)
		Yes	17188	(15.5%)
	Parental cancer	No	75798	(68.5%)
		Yes	34801	(31.5%)
	Parental diabetes	No	92312	(83.5%)
		Yes	18287	(16.5%)
	Parental high blood pressure	No	63987	(57.9%)
		Yes	46612	(42.1%)
	Parental bronchitis	No	95549	(86.4%)
		Yes	15050	(13.6%)
	Parental heart disease	No	65890	(59.6%)
		Yes	44709	(40.4%)
	Parental stroke	No	83089	(75.1%)
		Yes	27510	(24.9%)
**Lifestyle - sports**	Moderate (physical activity)	No or rarely	56022	(50.7%)
		Sometimes	54577	(49.3%)
	Vigorous (physical activity)	No	38412	(34.7%)
		Sometimes	72187	(65.3%)
	Walking (physical activity)	Sometimes	58833	(53.2%)
		Always	51766	(46.8%)
	Walking (pleasure)	No	28347	(25.6%)
		Yes	82252	(74.4%)
	Exercises (pleasure)	No	52351	(47.3%)
		Yes	58248	(52.7%)
	Sports (strenuous)	No	97489	(88.1%)
		Yes	13110	(11.9%)
	Light DIY	No	47283	(42.8%)
		Yes	63316	(57.2%)
	Heavy DIY	No	58310	(52.7%)
		Yes	52289	(47.3%)

Supplementary Table S2. **UK Biobank population descriptors II.**

Group	Variable	Category	Frequency	
**Alcohol/tobacco consumption**	Tobacco smoking	Never	43017	(38.9%)
		Sometimes	61341	(55.5%)
		Frequently	6241	(5.6%)
	Alcohol intake	Rarely	29929	(27.1%)
		Regularly	55154	(49.9%)
		Daily	25516	(23.1%)
**Diet and metabolism**	Dietary change	No	69393	(62.7%)
		Yes	41206	(37.3%)
	Weight change	No	63757	(57.6%)
		Yes - gained weight	29065	(26.3%)
		Yes - lost weight	17777	(16.1%)
	Metabolic rate	Lower than average	22071	(20.0%)
		Average	62813	(56.8%)
		High than average	25715	(23.3%)
	Body fat	Normal	50188	(45.4%)
		High	60411	(54.6%)
	Obesity	No	86175	(77.9%)
		Yes	24424	(22.1%)
	Diastolic (blood pressure)	Normal	48256	(43.6%)
		Prehypertension	39610	(35.8%)
		Hypertension I.	18212	(16.5%)
		Hypertension II.	4521	(4.1%)
	Pulse rate	Low	24537	(22.2%)
		Normal	85063	(76.9%)
		High	999	(0.9%)
	Systolic (blood pressure)	Normal	18872	(17.1%)
		Prehypertension	46614	(42.1%)
		Hypertension I.	32598	(29.5%)
		Hypertension II.	12515	(11.3%)
**General**	Age	40-50	34089	(30.8%)
		51-60	38893	(35.2%)
		61-70	37617	(34.0%)
	Sex	Female	60439	(54.6%)
		Male	50160	(45.4%)
	Handedness	Right	98166	(88.8%)
		Left	10646	(9.6%)
		Both	1787	(1.6%)

Supplementary Table S3. UK Biobank population descriptors III.

Group	Variable	Category	Frequency	
**Pain**	Headache	No	90674	(82.0%)
		Yes - recent	10575	(9.6%)
		Yes - chronic	9350	(8.5%)
	Facial pain	No	108983	(98.5%)
		Yes - recent	844	(0.8%)
		Yes - chronic	772	(0.7%)
	Neck/shoulder pain	No	88565	(80.1%)
		Yes - recent	7037	(6.4%)
		Yes - chronic	14997	(13.6%)
	Back pain	No	85359	(77.2%)
		Yes - recent	8114	(7.3%)
		Yes - chronic	17126	(15.5%)
	Stomach/abdominal pain	No	102769	(92.9%)
		Yes - recent	3671	(3.3%)
		Yes - chronic	4159	(3.8%)
	Hip pain	No	99601	(90.1%)
		Yes - recent	2567	(2.3%)
		Yes - chronic	8431	(7.6%)
	Knee pain	No	89080	(80.5%)
		Yes - recent	4768	(4.3%)
		Yes - chronic	16751	(15.1%)
	Pain allover	No	109441	(99.0%)
		Yes - recent	234	(0.2%)
		Yes - chronic	924	(0.8%)

Supplementary Table S4. UK Biobank population descriptors IV.

**Variable**	**Description**	**UKB ID**	**UKB field description**	**Details of derivation protocol**
Sex	Sex of subjects	31-0.0	Sex	None
Age	Categorized age	21003-0.0	Age when attended assessment centre	Variable 21003-0.0 was recoded into three groups having approximately equal number of subjects:<br>(0) subjects with age below or equal to 52 IF 21003-0.0 <= 52.<br>(1) subjects aged between 53 and 61 IF 21003-0.0 > 52 AND 21003-0.0 <= 61.<br>(2) subjects age above 61.
Townsend	Categorized Townsend deprivation index	189-0.0	Townsend deprivation index at recruitment	Variable 189-0.0 was recoded into three categories based on the distribution of values in the analyzed data:<br>(0) Deprived IF 189-0.0 <= −1.<br>(1) Mean IF 189-0.0 > −1 AND 189-0.0 <= 1.<br>(2) Affluent IF 189-0.0 > 1.
Qualification	College or university degree	6138-0.0<br>6138-0.1<br>6138-0.2<br>6138-0.3<br>6138-0.4<br>6138-0.5	Qualifications	Based on variables 6138-0.0, 6138-0.1, 6138-0.2, 6138-0.3, 6138-0.4, 6138-0.5 a new categorical variable was created indicating a college and/or university degree.<br>(0) No IF code 1 (college and/or university degree) is not present among the qualifications of a subject.<br>(1) YES IF code 1 (college and/or university degree) is present among the qualifications of a subject.
Household income	Categorized household income	738-0.0	Average total household income before tax	None
Ethnicity	Ethnic background	21000-0.0	Ethnic background	Variable 21000-0.0 was recoded to a binary variable as:<br>(0) White ethnic background.<br>(1) African, Asian or mixed ethnic background.

Supplementary Table S5. **UK Biobank variable transformations I.**

Variable	Description	UKB ID	UKB field description	Details of derivation protocol
Moderate physical	Frequency of moderate physical activity (recoded into 2 categories)	884-0.0	Number of days/week of moderate physical activity 10+ minutes	Variable 884-0.0 was recoded to a binary variable as:<br>(0) Sometimes IF 884-0.0 <= 3.<br>(1) Regurlarly IF 884-0.0 >= 4.
Vigorous physical	Frequency of vigorous physical activity (recoded into 2 categories)	904-0.0	Number of days/week of vigorous physical activity 10+ minutes	Variable 904-0.0 was recoded to a binary variable as:<br>(0) Never IF 904-0.0 = 0.<br>(1) Sometimes or frequently IF 904-0.0 >= 1.
Walking physical	Frequency of walking (recoded into 2 categories)	864-0.0	Number of days/week walked 10+ minutes	Variable 864-0.0 was recoded to a binary variable as:<br>(0) Sometimes IF 864-0.0 <= 6.<br>(1) Everyday IF 864-0.0 = 7.
Walking	Walking for pleasure (not as a means of transport)	6164-0.0<br>6164-0.1<br>6164-0.2<br>6164-0.3<br>6164-0.4	Types of physical activity in last 4 weeks	Based on array variables 6164-0.0, 6164-0.1, 6164-0.2, 6164-0.3, 6164-0.4, the presence of Walking for pleasure item (code: 1) was examined and a corresponding binary variable was created:<br>(0) No.<br>(1) Yes.
Exercises	Other exercises (eg: swimming, cycling, keep fit, bowling)	6164-0.0<br>6164-0.1<br>6164-0.2<br>6164-0.3<br>6164-0.4	Types of physical activity in last 4 weeks	Based on array variables 6164-0.0, 6164-0.1, 6164-0.2, 6164-0.3, 6164-0.4, the presence of Other exercises item (code: 2) was examined and a corresponding binary variable was created:<br>(0) No.<br>(1) Yes.
Sports	Strenuous sports	6164-0.0<br>6164-0.1<br>6164-0.2<br>6164-0.3<br>6164-0.4	Types of physical activity in last 4 weeks	Based on array variables 6164-0.0, 6164-0.1, 6164-0.2, 6164-0.3, 6164-0.4, the presence of Strenuous sports (code: 3) item was examined and a corresponding binary variable was created:<br>(0) No.<br>(1) Yes.
Light DIY	Light DIY (e.g.: pruning, watering the lawn)	6164-0.0<br>6164-0.1<br>6164-0.2<br>6164-0.3<br>6164-0.4	Types of physical activity in last 4 weeks	Based on array variables 6164-0.0, 6164-0.1, 6164-0.2, 6164-0.3, 6164-0.4, the presence of Light DIY item (code: 4) was examined and a corresponding binary variable was created:<br>(0) No.<br>(1) Yes.
Heavy DIY	Heavy DIY (e.g.: weeding, lawn mowing, carpentry, digging)	6164-0.0<br>6164-0.1<br>6164-0.2<br>6164-0.3<br>6164-0.4	Types of physical activity in last 4 weeks	Based on array variables 6164-0.0, 6164-0.1, 6164-0.2, 6164-0.3, 6164-0.4, the presence of Heavy DIY item (code: 5) was examined and a corresponding binary variable was created:<br>(0) No.<br>(1) Yes.

Supplementary Table S6. **UK Biobank variable transformations II.**

Variable	Description	UKB ID	UKB field description	Details of derivation protocol
Tobacco smoking	Tobacco smoking (recoded)	1239-0.0<br>1249-0.0	Current and past tobacco smoking	Based on items 1239-0.0 and 1249-0.0 an aggregate variable describing tobacco smoking was created with the following categories:<br>(0) Never IF 1249-0.0 = 4 (Never smoked).<br>(1) Sometimes IF 1249-0.0 <> 4 (Subject used to smoke regularly or just occasionally) AND 1239-0.0 <> 1 (Currently not smoking or only occasionally).<br>(2) Currently yes IF 1239-0.0 = 1 (Smoking on most or all days).
Dietary change	Major dietary changes in the last 5 years (binarized)	1538-0.0	Major dietary changes in the last 5 years	Variable 1538-0.0 was recoded into two categories:<br>(0) No (No major changes) IF 1538-0.0 = 0.<br>(1) Yes (Because of illness or other reasons) IF 1538-0.0 = 1 or 2.
Alcohol intake	Alcohol intake frequency (recoded)	1558-0.0	Alcohol intake frequency	Variable 1558-0.0 was recoded into three categories:<br>(0) Rarely IF 1558-0.0 >= 4 (One to three times a month or Special occasions only or never).<br>(1) Regularly IF 1558-0.0 = 2 or 3 (Three or four times a week or Once or twice a week).<br>(2) Daily IF 1558-0.0 = 1 (Daily or almost daily).

Supplementary Table S7. UK Biobank variable transformations III.

Variable	Description	UKB ID	UKB field description	Details of derivation protocol
Breastfed	Breastfed as a baby (yes/no)	1677-0.0	Breastfed as a baby	None
Body size	Comparative body size at age 10	1687-0.0	Comparative body size at age 10	None
First intercourse	Age of first sexual intercourse (categorized)	2139-0.0	Age first had sexual intercourse	Variable 2139-0.0 was recoded into three categories:<br>(0) Under 14 IF 2139-0.0 $<$ 14.<br>(1) Between 14 and 16 IF 2139-0.0 $>=$ 14 AND 2139-0.0 $<=$ 16.<br>(2) Above 16 IF 2139-0.0 $>$ 16.
Height size	Comparative height size at age 10	1697-0.0	Comparative height size at age 10	None
Hand	Handedness (chirality/laterality)	1707-0.0	Handedness (chirality/laterality)	None
Multiple birth	Part of a multiple birth (yes/no)	1777-0.0	Part of a multiple birth	None
Maternal smoking	Maternal smoking around birth (yes/no)	1787-0.0	Maternal smoking around birth	None

Supplementary Table S8. **UK Biobank variable transformations - Childhood related factors**

Variable	Description	UKB ID	UKB field description	Details of derivation protocol
Current depressive symptoms	Current depressive symptoms score	2050-0.0	Frequency of depressed mood in last 2 weeks	Based on the sum of items 2050-0.0, 2060-0.0, 2070-0.0, 2080-0.0 divided by the number of items present (score range: 1-4);<br>(0) None IF score is 1.<br>(1) Mild IF score > 1 and < 2.<br>(2) Severe IF score $>= 2$.
		2060-0.0	Frequency of unenthusiasm or disinterest in last 2 weeks	
		2070-0.0	Frequency of tenseness or restlessness in last 2 weeks	
		2080-0.0	Frequency of tiredness or lethargy in last 2 weeks	
Neuroticism	Derived neuroticism score (UKB 20127)	1920-0.0	Mood swings	Based on the sum of neuroticsm items 1920-0.0, 1930-0.0, 1940-0.0, 1950-0.0, 1960-0.0, 1970-0.0, 1980-0.0, 1990-0.0, 2000-0.0, 2010-0.0, 2020-0.0, 2030-0.0 divided by the number of non-missing neuroticsm items a derived neuroticsm score is computed (score range: 0-1). This score is then classified as:<br>(0) Low IF score ≥ 0 and < 0.4.<br>(1) Medium IF score ≥ 0.4 and < 0.6.<br>(2) High IF score ≥ 0.6 and ≤ 1.
		1930-0.0	Miserableness	
		1940-0.0	Irritability	
		1950-0.0	Sensitivity or hurt feelings	
		1960-0.0	Fed-up feelings	
		1970-0.0	Nervous feelings	
		1980-0.0	Worrier or anxious feelings	
		1990-0.0	Tense or 'highly strung'	
		2000-0.0	Worry too long after embarrassment	
		2010-0.0	Suffer from 'nerves'	
		2020-0.0	Loneliness, isolation	
		2030-0.0	Guilty feelings	
Manic depression	Bipolar disorder or manic depression	20002-0.0-20002-0.28	Non-cancer illness code, self-reported	Decoded from non-cancer illness codes using code= 1291 (binary descriptor)
Depression	Lifetime depression, self reported	20002-0.0 20002-0.28	Non-cancer illness code, self-reported	Decoded from non-cancer illness codes using code= 1286 (binary descriptor)

Supplementary Table S9. **UKB variable transformations - Mental health variables I.**

Variable	Description	UKB ID	UKB field description	Details of derivation protocol
Insomnia	Sleeplessness or insomnia	1200-0.0	Sleeplessness or insomnia	None
Satisfaction	Satisfaction score	4526-0.0	Happiness	Based on the sum of items 4526-0.0, 4537-0.0, 4548-0.0, 4559-0.0, 4570-0.0, 4581-0.0. divided by the number of non-missing items (score range: 0-6). This score is categorized as:<br>(0) High IF score < 2.<br>(1) Average IF score >= 2 and < 3.<br>(2) Low IF score >= 3.
		4537-0.0	Work/job satisfaction	
		4548-0.0	Health satisfaction	
		4559-0.0	Family relationship satisfaction	
		4570-0.0	Friendships satisfaction	
		4581-0.0	Financial situation satisfaction	
Risk taking	Risk taking (binary descriptor)	2040-0.0	Risk taking	None
Life stress	Recent life events in the last 2 years	6145-0.0<br>6145-0.1<br>6145-0.2<br>6145-0.3<br>6145-0.4<br>6145-0.5	Illness, injury, bereavement, stress in last 2 years	Based on the number of life stress items present (6145-0.0 -6145-0.5);<br>(0) None IF no items are present.<br>(1) Low IF 1 item is present.<br>(2) Moderate or High IF >= 2 items are present.

Supplementary Table S10. **UKB variable transformations - Mental health variables II.**

Variable	Description	UKB ID	UKB field description	Details of derivation protocol
Visits	Frequency of friend or family visits	1031-0.0	Frequency of friend/family visits	Variable 1031-0.0 is recoded to three categories as:<br>(0) No or rarely IF 1031-0.0 >= 4.<br>(1) Weekly IF 1031-0.0 = 3.<br>(2) Frequently IF 1031-0.0 <= 2.
Social activity	Subject participates in any social activity	6160-0.0<br>6160-0.1<br>6160-0.2<br>6160-0.3<br>6160-0.4	Leisure or social activities	Leisure or social activity items such as (1) Sports club or gym, (2) Pub or social club, (3) Religious group, (4) Adult education class, (5) Other group activity were collected and aggregated for each subject from array variables 6160-0.0, 6160-0.1, 6160-0.2, 6160-0.3, 6160-0.4. Then subjects were classified as:<br>(0) No (subject participates in no leisure/social activities) IF none of the above items appeared in array 6160.<br>(1) Yes (subject participates in some leisure/social activities) IF at least one of the above items appeared in array 6160.
Confide	Subject is able to confide in someone	2110-0.0	Able to confide	Variable 2110-0.0 is recoded to three categories as:<br>(0) Never or almost never IF 2110-0.0 = 0.<br>(1) Sometimes IF 2110-0.0 was between 1 and 4.<br>(2) Almost daily IF 2110-0.0 = 5.

Supplementary Table S11. **UKB variable transformations - Social activity variables**

Variable	Description	UKB ID	UKB field description	Details of derivation protocol
Pain headache	Headache (none, recent, chronic)	6159-0.0<br>6159-0.1<br>6159-0.2<br>6159-0.3<br>6159-0.4<br>6159-0.5<br>6159-0.6	Pain type(s) experienced in last month	Based on pain type variables 6159-0.0, 6159-0.1, 6159-0.2, 6159-0.3, 6159-0.4, 6159-0.5, 6159-0.6 and 3799-0.0 a categorical variable describing the presence of headache was created.<br>(0) No IF Pain type not present (6159-0.0 - 6159-0.6).<br>(1) Yes-recent IF Pain type present (6159-0.0 - 6159-0.6) AND Pain type not present for 3+ months (3799-0.0).<br>(2) Yes-3M IF Pain type present (6159-0.0 - 6159-0.6) AND Pain type present for 3+ months (3799-0.0).
		3799-0.0	Headaches for 3+ months	
Pain facial	Facial pain (none, recent, chronic)	6159-0.0<br>6159-0.1<br>6159-0.2<br>6159-0.3<br>6159-0.4<br>6159-0.5<br>6159-0.6	Pain type(s) experienced in last month	Based on pain type variables 6159-0.0, 6159-0.1, 6159-0.2, 6159-0.3, 6159-0.4, 6159-0.5, 6159-0.6 and 3799-0.0 a categorical variable describing the presence of facial pain was created.<br>(0) No IF Pain type not present (6159-0.0 - 6159-0.6);<br>(1) Yes-recent IF Pain type present (6159-0.0 - 6159-0.6) AND Pain type not present for 3+ months (4067-0.0);<br>(3) Yes-3M IF Pain type present (6159-0.0 - 6159-0.6) AND Pain type present for 3+ months (4067-0.0)
		4067-0.0	Facial pains for 3+ months	
Pain neck-shoulder	Neck or shoulder pain (none, recent, chronic)	6159-0.0<br>6159-0.1<br>6159-0.2<br>6159-0.3<br>6159-0.4<br>6159-0.5<br>6159-0.6	Pain type(s) experienced in last month	Based on pain type variables 6159-0.0, 6159-0.1, 6159-0.2, 6159-0.3, 6159-0.4, 6159-0.5, 6159-0.6 and 3799-0.0 a categorical variable describing the presence of neck or shoulder pain was created.<br>(0) No IF Pain type not present (6159-0.0 - 6159-0.6).<br>(1) Yes-recent IF Pain type present (6159-0.0 - 6159-0.6) AND Pain type not present for 3+ months (3404-0.0).<br>(2) Yes-3M IF Pain type present (6159-0.0 - 6159-0.6) AND Pain type present for 3+ months (3404-0.0).
		3404-0.0	Neck/shoulder pain for 3+ months	

Supplementary Table S12. **UKB variable transformations - Pain variables I.**

Variable	Description	UKB ID	UKB field description	Details of derivation protocol
Pain back	Back pain (none, recent, chronic)	6159-0.0 6159-0.1 6159-0.2 6159-0.3 6159-0.4 6159-0.5 6159-0.6	Pain type(s) experienced in last month	Based on pain type variables 6159-0.0, 6159-0.1, 6159-0.2, 6159-0.3, 6159-0.4, 6159-0.5, 6159-0.6 and 3571-0.0 a categorical variable describing the presence of back pain was created.<br>(0) No IF Pain type not present (6159-0.0 - 6159-0.6).<br>(1) Yes-recent IF Pain type present (6159-0.0 - 6159-0.6) AND Pain type not present for 3+ months (3571-0.0).<br>(2) Yes-3M IF Pain type present (6159-0.0 - 6159-0.6) AND Pain type present for 3+ months (3571-0.0).
		3571-0.0	Back pain for 3+ months	
Pain stomach or abdominal	Stomach or abdominal pain (none, recent, chronic)	6159-0.0 6159-0.1 6159-0.2 6159-0.3 6159-0.4 6159-0.5 6159-0.6	Pain type(s) experienced in last month	Based on pain type variables 6159-0.0, 6159-0.1, 6159-0.2, 6159-0.3, 6159-0.4, 6159-0.5, 6159-0.6 and 3471-0.0 a categorical variable describing the presence of stomach pain was created.<br>(0) No IF Pain type not present (6159-0.0 - 6159-0.6).<br>(1) Yes-recent IF Pain type present (6159-0.0 - 6159-0.6) AND Pain type not present for 3+ months (3471-0.0).<br>(2) Yes-3M IF Pain type present (6159-0.0 - 6159-0.6) AND Pain type present for 3+ months (3471-0.0).
		3741-0.0	Stomach or abdominal pain for 3+ months	
Pain hip	Hip pain (none, recent, chronic)	6159-0.0 6159-0.1 6159-0.2 6159-0.3 6159-0.4 6159-0.5 6159-0.6	Pain type(s) experienced in last month	Based on pain type variables 6159-0.0, 6159-0.1, 6159-0.2, 6159-0.3, 6159-0.4, 6159-0.5, 6159-0.6 and 3414-0.0 a categorical variable describing the presence of hip pain was created.<br>(0) No IF Pain type not present (6159-0.0 - 6159-0.6).<br>(1) Yes-recent IF Pain type present (6159-0.0 - 6159-0.6) AND Pain type not present for 3+ months (3414-0.0).<br>(2) Yes-3M IF Pain type present (6159-0.0 - 6159-0.6) AND Pain type present for 3+ months (3414-0.0).
		3414-0.0	Hip pain for 3+ months	

Supplementary Table S13. **UKB variable transformations - Pain variables II.**

Variable	Description	UKB ID	UKB field description	Details of derivation protocol
Pain knee	Knee pain (none, recent, chronic)	6159-0.0 6159-0.1 6159-0.2 6159-0.3 6159-0.4 6159-0.5 6159-0.6	Pain type(s) experienced in last month	Based on pain type variables 6159-0.0, 6159-0.1, 6159-0.2, 6159-0.3, 6159-0.4, 6159-0.5, 6159-0.6 and 3773-0.0 a categorical variable describing the presence of knee pain was created.<br>(0) No IF Pain type not present (6159-0.0 - 6159-0.6).<br>(1) Yes-recent IF Pain type present (6159-0.0 - 6159-0.6) AND Pain type not present for 3+ months (3773-0.0).<br>(2) Yes-3M IF Pain type present (6159-0.0 - 6159-0.6) AND Pain type present for 3+ months (3773-0.0).
		3773-0.0	Knee pain for 3+ months	
Pain allover	Pain all over the body (none, recent, chronic)	6159-0.0 6159-0.1 6159-0.2 6159-0.3 6159-0.4 6159-0.5 6159-0.6	Pain type(s) experienced in last month	Based on pain type variables 6159-0.0, 6159-0.1, 6159-0.2, 6159-0.3, 6159-0.4, 6159-0.5, 6159-0.6 and 2956-0.0 a categorical variable describing general pain was created.<br>(0) No IF Pain type not present (6159-0.0 - 6159-0.6).<br>(1) Yes-recent IF Pain type present (6159-0.0 - 6159-0.6) AND Pain type not present for 3+ months (2956-0.0).<br>(2) Yes-3M IF Pain type present (6159-0.0 - 6159-0.6) AND Pain type present for 3+ months (2956-0.0).
		2956-0.0	General pain for 3+ months	
Falls	Subject fell at least once in the last year (yes/no)	2296-0.0	Falls in the last year	None

Supplementary Table S14. **UKB variable transformations - Pain variables III.**

Variable	Description	UKB ID	UKB field description	Details of derivation protocol
Diastolic	Diastolic blood pressure	4079-0.0<br>4079-0.1	Diastolic blood pressure	Based on mean value of 4079-0.0 and 4079-0.1 a categorical variable was created using cut-off values of the JNC7* guidelines:<br>(0) Normal IF mean diastolic blood pressure < 80.<br>(1) Prehypertension IF mean diastolic blood pressure >= 80 AND < 90.<br>(2) Hypertension stage I. IF mean diastolic blood pressure >= 90 AND < 100.<br>(3) Hypertension stage II. IF mean diastolic blood pressure >= 100.<br>*https://www.nhlbi.nih.gov/files/docs/guidelines/express.pdf
Systolic	Systolic blood pressure	4080-0.0<br>4080-0.1	Systolic blood pressure, automated reading	Based on the mean value of 4080-0.0 and 4080-0.1 a categorical variable was created using cut-off values of the JNC7* guidelines:<br>(0) Normal IF mean value < 120.<br>(1) Prehypertension IF mean value >= 120 AND < 140.<br>(2) Hypertension stage I. IF mean value >= 140 AND < 160.<br>(3) Hypertension stage II. IF mean value >= 160.<br>*https://www.nhlbi.nih.gov/files/docs/guidelines/express.pdf
Pulse rate	Pulse rate	102-0.0<br>102-0.1	Pulse rate, automated reading	Based on mean value of 102-0.0 and 102-0.1 a categorical variable was created using cut-off values of the JNC7 guidelines:<br>(0) Low IF mean value < 60.<br>(1) Normal IF mean value >= 61 AND < 100.<br>(2) High IF mean value >= 100.

Supplementary Table S15. UKB variable transformations - Blood pressure variables

Variable	Description	UKB ID	UKB field description	Details of derivation protocol
Obesity	Obesity based on body mass index (BMI)	21001-0.0	Body mass index (BMI)	Variable 21001-0.0 was recoded using the following categories*:<br>(0) No IF 21001-0.0 < 30.<br>(2) Yes IF 21001-0.0 >= 30.<br>*based on WHO guidlines https://www.who.int/topics/obesity/en/
Metabolic rate	Basal metabolic rate	23105-0.0	Basal metabolic rate	Variable 23099-0.0 was recoded into 3 categories based on gender:<br>(0) Lower than average IF Male AND (23099-0.0 < 7000) OR IF Female AND (23099-0.0 < 5100).<br>(1) Average IF Male AND (23099-0.0 >= 7000 and < 8500) OR IF Female AND (23099-0.0 >= 5100 AND < 6000).<br>(2) Higher than average IF Male AND (23099-0.0 >= 8500) OR IF Female AND (23099-0.0 >= 6000).
Body fat	Body fat percentage	23099-0.0	Body fat percentage	Variable 23099-0.0 was recoded into the following categories based on gender*:<br>(0) Normal IF Female AND 23099-0.0 < 35% OR IF Male AND 23099-0.0 < 25%<br>(1) High IF Female AND 23099-0.0 >= 35% OR IF Male AND 23099-0.0 >= 25%<br>*using WHO guidlines http://whqlibdoc.who.int/trs/WHO_TRS_854.pdf

Supplementary Table S16. **UKB variable transformations - Metabolic variables**

Variable	Category	OR	CI 95%		Variable	Category	OR	CI 95%	
**Mental health**					**Financial background**				
Bipolar disorder	No				Townsend	Affluent			
	Yes	1.92	1.3	2.81		Mean	1.14	1.06	1.23
Current depressive symptoms	No					Deprived	1.56	1.46	1.66
	Mild	2.41	2.22	2.61	Qualification (Higher education)	No			
	Severe	10.66	9.85	11.53		Yes	1.10	1.05	1.16
Insomnia	Never				Household income	Category I.			
	Sometimes	1.55	1.44	1.68		Category II.	0.64	0.59	0.69
	Usually	2.45	2.27	2.65		Category III.	0.56	0.52	0.6
Neuroticism	Low					Category IV.	0.42	0.38	0.45
	Medium	3.44	3.18	3.71		Category V.	0.34	0.3	0.39
	High	9.26	8.64	9.91	**Parental illnesses**				
Risk taking	No				Parental depression	No			
	Yes	1.07	1.01	1.14		Yes	2.84	2.66	3.04
Satisfaction	High				Parental Parkinson	No			
	Average	1.71	1.56	1.86		Yes	1.01	0.88	1.15
	Low	4.22	3.84	4.63	Parental Alzheimer's	No			
**Social factors**						Yes	1.06	0.99	1.14
Social activity	No				Parental cancer	No			
	Yes	1.28	1.21	1.36		Yes	0.98	0.92	1.04
Visits friends and family	No or rarely				Parental diabetes	No			
	Weekly	0.88	0.82	0.95		Yes	1.12	1.05	1.2
	Frequently	1.00	0.93	1.07	Parental high blood pressure	No			
Confide	Never or rarely					Yes	1.20	1.14	1.27
	Sometimes	0.91	0.84	0.98	Parental bronchitis	No			
	Frequently	0.61	0.56	0.66		Yes	1.26	1.17	1.35
**Childhood factors**					Parental heart disease	No			
Body size (childhood)	Thin					Yes	1.17	1.11	1.24
	Plump	1.17	1.09	1.26	Parental stroke	No			
	Average	0.90	0.85	0.96		Yes	1.02	0.96	1.09
Height size (childhood)	Short				**Lifestyle - sports**				
	Tall	0.95	0.88	1.02	Moderate (physical activity)	No or rarely			
	Average	0.90	0.84	0.96		Sometimes	0.82	0.78	0.86
Breastfed	No				Vigorous (physical activity)	No			
	Yes	0.84	0.79	0.89		Sometimes	0.70	0.66	0.74
Maternal smoking	No				Walking (physical activity)	Sometimes			
	Yes	1.15	1.09	1.22		Always	0.85	0.8	0.89
First intercourse	< 14				Walking (pleasure)	No			
	14 – 16	0.61	0.51	0.72		Yes	0.75	0.71	0.8
	> 16	0.48	0.41	0.56	Exercises (pleasure)	No			
**Life stress**						Yes	0.68	0.65	0.72
Life stress	None				Sports (strenuous)	No			
	1 - event	1.53	1.44	1.62		Yes	0.55	0.50	0.61
	2 or more events	2.59	2.41	2.78	Light DIY	No			
Falls	None					Yes	0.87	0.83	0.92
	Only 1	1.58	1.47	1.7	Heavy DIY	No			
	More than 1	2.75	2.53	2.98		Yes	0.71	0.67	0.75

Supplementary Table S17. Univariate effect size of UKB variables with respect to lifetime depression 1. Odds ratios (OR) for each variable are computed using the lowest category as basis. CI 95% denotes the lower and upper thresholds of the confidence interval of odds ratios.

Variable	Category	OR	CI 95%	
**Alcohol and tobacco consumption**				
Tobacco smoking	Never			
	Sometimes	1.14	1.08	1.21
	Frequently	1.87	1.69	2.06
Alcohol intake	Rarely			
	Regularly	0.57	0.54	0.61
	Daily	0.62	0.57	0.66
**Diet and metabolism**				
Dietary change	No			
	Yes	1.51	1.44	1.60
Weight change	No			
	Yes - gained weight	1.88	1.78	2.00
	Yes - lost weight	1.57	1.46	1.69
Metabolic rate	Low			
	Moderate	1.10	1.03	1.19
	High	1.58	1.46	1.72
Body fat	Normal			
	High	1.56	1.48	1.65
Obesity	No			
	Yes	1.60	1.51	1.70
Diastolic (blood pressure)	Normal			
	Prehypertension	0.89	0.84	0.95
	Hypertension I.	0.86	0.79	0.92
	Hypertension II.	0.95	0.83	1.09
Systolic (blood pressure)	Normal			
	Prehypertension	0.80	0.75	0.86
	Hypertension I.	0.66	0.61	0.72
	Hypertension II.	0.51	0.45	0.56
Pulse rate	Low			
	Normal	1.25	1.17	1.33
	High	1.58	1.22	2.03

Variable	Category	OR	CI 95%	
**Pain**				
Headache	No			
	Yes - recent	1.86	1.72	2.01
	Yes - chronic	2.52	2.34	2.71
Facial pain	No			
	Yes - recent	2.23	1.79	2.77
	Yes - chronic	3.00	2.44	3.68
Neck or shoulder pain	No			
	Yes - recent	1.51	1.37	1.67
	Yes - chronic	1.94	1.82	2.07
Back pain	No			
	Yes - recent	1.28	1.16	1.42
	Yes - chronic	1.90	1.79	2.03
Stomach or abdominal pain	No			
	Yes - recent	1.76	1.56	1.99
	Yes - chronic	2.39	2.16	2.64
Hip pain	No			
	Yes - recent	1.54	1.33	1.79
	Yes - chronic	1.86	1.72	2.02
Knee pain	No			
	Yes - recent	1.31	1.16	1.48
	Yes - chronic	1.42	1.33	1.52
Pain allover	No			
	Yes - recent	1.72	1.08	2.72
	Yes - chronic	3.12	2.59	3.75
**General**				
Age	40-50			
	51-60	0.89	0.84	0.95
	61-70	0.63	0.59	0.68
Sex	Female			
	Male	0.6	0.57	0.63
Handedness	Right			
	Left	0.96	0.87	1.05
	Both	1.17	0.96	1.42

Supplementary Table S18. Univariate effect size of UKB variables with respect to lifetime depression 2. Odds ratios (OR) for each variable are computed using the lowest category as basis. CI 95% denotes the lower and upper thresholds of the confidence interval of odds ratios.

Predictive power of relevant variable sets

Variable sets		Residual variance			Predictive performance			Cross-entropy	
		Score	RRV	Rank	Score	Ratio	Rank	Score	Rank
MBS-1	Current depressive symptoms, Neuroticism, Parental depression, Body fat, Bipolar disorder, Risk taking, Sex	0.190	24.1%	5	0.721	99.7%	3	2.574	2
MBS-2	Current depressive symptoms, Neuroticism, Parental depression, Body fat, Bipolar disorder, Qualification, Parental bronchitis	0.182	27.3%	1	0.710	98.3%	5	2.575	3
MBS-3	Current depressive symptoms, Neuroticism, Parental depression, Body fat, Bipolar disorder, Parental bronchitis, Parental Alzheimer's, Exercises, Sports, Moderate physical, Age, Alcohol intake, Household income	0.183	26.8%	2	0.731	101.1%	1	2.525	1
MBS-4	Current depressive symptoms, Neuroticism, Parental depression, Life stress, Bipolar disorder, Maternal smoking, Parental Alzheimer's	0.188	24.7%	4	0.717	99.2%	4	2.598	5
Direct	Current depressive symptoms, Neuroticism, Parental depression, Body fat	0.188	24.8%	3	0.723	100%	2	2.588	4

Supplementary Table S19. Predictive measures of sets of relevant variables with respect to lifetime depression. *MBS-1-4* denote the top four sets of relevant variables (Markov blanket sets) with the highest probability. *Direct* denotes the set of directly relevant variables with the highest probability. *Score* denotes the computed value of the corresponding measure. *RRV* stands for the reduction in residual variance compared to the residual variance of the random classifier. *Ratio* denotes the ratio of the predictive performance score of the given set of variables and that of the *Direct* set.

Synergistic effects

**Body fat (I.)**		**I. + Dietary change (II.)**				**I. + II. + Weight change (III.)**			
				$CI_{95\%}$				$CI_{95\%}$	
	CR-OR		CR-OR	Low	High		CR-OR	Low	High
Normal	0.64	No	0.58	0.54	0.62	No	0.53	0.5	0.58
						Gained weight	0.91	0.81	1.04
						Lost weight	0.76	0.64	0.90
		Yes	0.98	0.91	1.06	No	0.70	0.63	0.78
						Gained weight	1.60	1.39	1.84
						Lost weight	1.20	1.05	1.36
High	1.56	No	1.02	0.96	1.08	No	0.73	0.68	0.79
						Gained weight	1.45	1.34	1.56
						Lost weight	1.17	1.01	1.36
		Yes	1.69	1.59	1.79	No	1.23	1.13	1.34
						Gained weight	1.92	1.78	2.08
						Lost weight	1.47	1.33	1.62

Supplementary Table S20. Synergistic effects of Body fat, Dietary change, and Weight change with respect to lifetime depression. CR-OR and $CI_{95\%}$ denotes the configuration relative odds ratio and its 95% confidence interval respectively.

Parametric interactions

Based on the pairwise structural interaction between household income, alcohol intake, age and physical activity (sports), we investigate the multivariate effect size of these variables. Parametric measures shown in Supplementary Table S21, Supplementary Figure S1, and Supplementary Figure S2 indicate that subpopulations created according to household income have considerably different configuration relative odds ratios (CR-OR) with respect to lifetime depression. The more affluent the subject is, the less likely is the presence of lifetime depression. Subjects of the least affluent group have a relatively high odds ratio (CR-OR: 1.91). Subjects in the low and medium household income groups have a neutral effect size with respect to lifetime depression (CR-OR: 1.08 and 0.9), whereas subjects of the high and very high income groups are more protected than the others (CR-OR: 0.63 and 0.55 respectively). When the effect of age is also observed, in each of the income subgroups subjects with a lower age had a higher chance of having a depression.

Furthermore, if the effect of alcohol consumption is also taken into account, rare alcohol consumption seems to carry the highest risk of susceptibility to depression followed by daily consumption, and finally the lowest risk is related to regular consumption. These effects are observable in case of subjects with moderate to very low income across all age subgroups. However, subjects with high or very high income do not strictly follow this pattern, particularly in case of subjects with age above 61. The two endpoints of *Household income - Age - Alcohol intake* variable configurations are (1) subjects with very low income, age below 53, rare alcohol consumption (CR-OR: 3.75), and (2) subjects with very high income, age above 61, rare alcohol consumption (CR-OR: 0.29). Note that in the latter segment the effect size related to regular alcohol consumption is similar (CR-OR: 0.31).

**Household income (I.)**		**I. + Age (II.)**		**I.+II.+ Alcohol intake (III.A)**				**I.+II.+ Sports (III.B)**			
						$CI_{95\%}$				$CI_{95\%}$	
					CR-OR	Low	High		CR-OR	Low	High
				Rarely	3.75	3.29	4.29				
				Regularly	2.13	1.80	2.52	No	3.15	2.84	3.49
		<53	3.04	Daily	2.93	2.19	3.92	Yes	1.90	1.32	2.74
				Rarely	2.57	2.26	2.92				
				Regularly	1.50	1.29	1.75	No	2.09	1.90	2.29
		53-61	2.02	Daily	1.75	1.41	2.18	Yes	0.95	0.59	1.53
				Rarely	1.34	1.18	1.53				
				Regularly	0.95	0.82	1.10	No	1.12	1.02	1.23
Very Low	1.91	61<	1.11	Daily	0.99	0.8	1.22	Yes	0.90	0.56	1.44
				Rarely	2.04	1.77	2.35				
				Regularly	1.25	1.08	1.46	No	1.78	1.61	1.97
		<53	1.65	Daily	1.82	1.43	2.31	Yes	0.79	0.55	1.14
				Rarely	1.55	1.34	1.78				
				Regularly	1.04	0.92	1.19	No	1.30	1.19	1.42
		53-61	1.24	Daily	1.18	0.98	1.42	Yes	0.54	0.35	0.84
				Rarely	0.88	0.75	1.03				
				Regularly	0.61	0.53	0.71	No	0.70	0.63	0.77
Low	1.08	61<	0.69	Daily	0.70	0.59	0.85	Yes	0.66	0.45	0.96
				Rarely	1.50	1.31	1.72				
				Regularly	0.99	0.87	1.11	No	1.31	1.20	1.44
		<53	1.20	Daily	1.31	1.08	1.60	Yes	0.70	0.55	0.90
				Rarely	1.28	1.10	1.49				
				Regularly	0.77	0.68	0.88	No	0.96	0.88	1.06
		53-61	0.94	Daily	0.98	0.82	1.16	Yes	0.75	0.56	1.01
				Rarely	0.96	0.79	1.18				
				Regularly	0.49	0.41	0.59	No	0.64	0.57	0.72
Medium	0.90	61<	0.61	Daily	0.59	0.48	0.72	Yes	0.26	0.14	0.47
				Rarely	0.97	0.81	1.16				
				Regularly	0.57	0.49	0.66	No	0.80	0.71	0.89
		<53	0.70	Daily	0.84	0.68	1.04	Yes	0.42	0.33	0.55
				Rarely	0.94	0.76	1.17				
				Regularly	0.69	0.59	0.79	No	0.74	0.66	0.83
		53-61	0.71	Daily	0.64	0.52	0.79	Yes	0.52	0.37	0.73
				Rarely	0.95	0.71	1.27				
				Regularly	0.53	0.41	0.68	No	0.57	0.49	0.68
High	0.63	61<	0.56	Daily	0.42	0.31	0.57	Yes	0.44	0.25	0.78
				Rarely	1.03	0.71	1.50				
				Regularly	0.53	0.40	0.68	No	0.74	0.60	0.90
		<53	0.61	Daily	0.59	0.41	0.84	Yes	0.34	0.22	0.53
				Rarely	0.79	0.47	1.33				
				Regularly	0.52	0.38	0.71	No	0.64	0.51	0.80
		53-61	0.57	Daily	0.58	0.40	0.83	Yes	0.31	0.16	0.60
				Rarely	0.29	0.09	0.91				
				Regularly	0.31	0.16	0.59	No	0.39	0.26	0.59
Very High	0.55	61<	0.39	Daily	0.52	0.31	0.87	Yes	0.38	0.12	1.19

Supplementary Table S21. Parametric interactions of Household income, Age, Alcohol intake and Sports with respect to lifetime depression. CR-OR and $CI_{95\%}$ denotes the configuration relative odds ratio and its 95% confidence interval respectively.

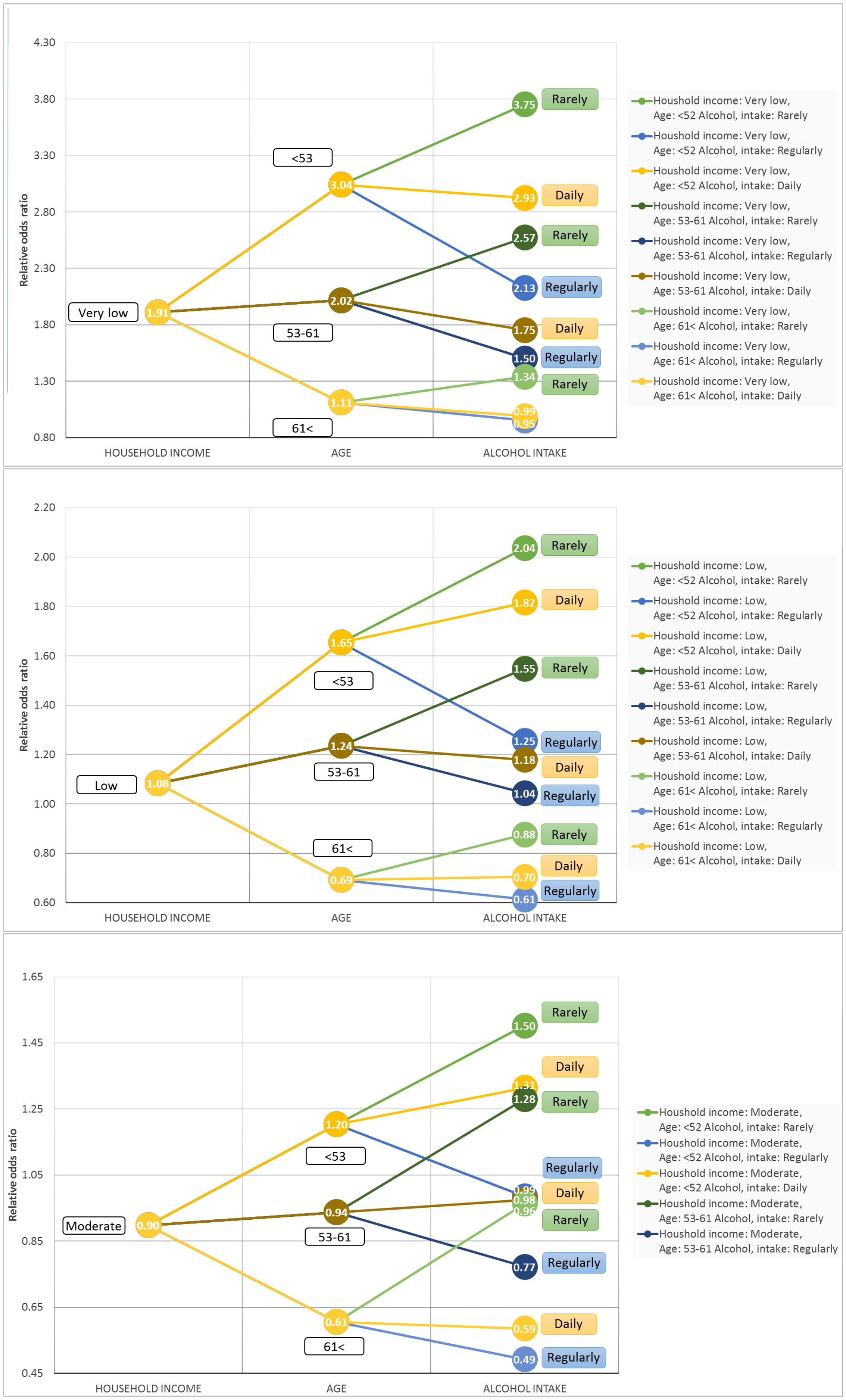

Supplementary Figure S1. **Parametric interactions of Household income, Age, and Alcohol intake with respect to lifetime depression.**

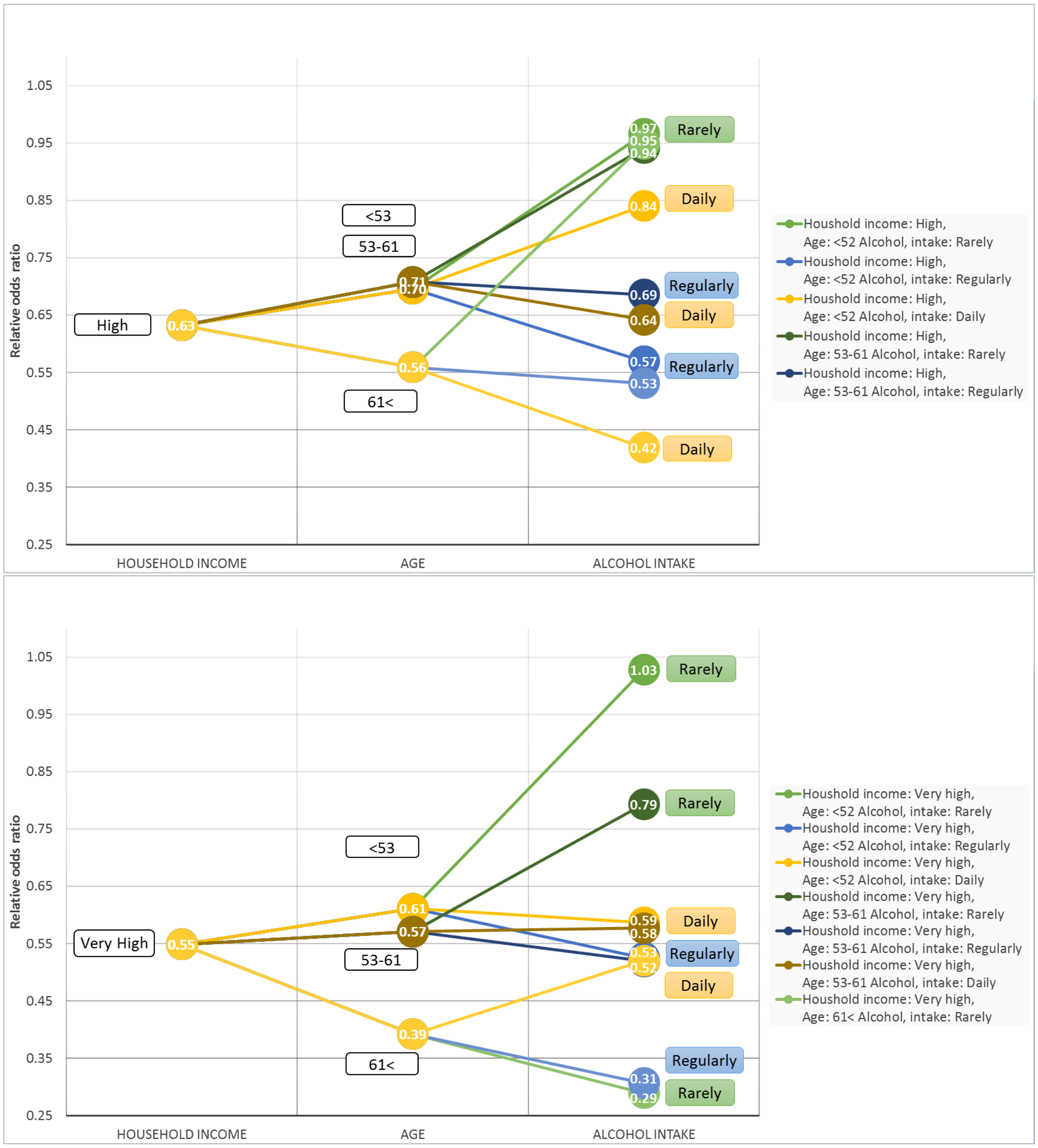

Supplementary Figure S2. Parametric interactions of Household income, Age, and Alcohol intake with respect to lifetime depression.

In addition, Supplementary Table S21 also displays the effect of doing strenuous sports in conjunction with household income and age. In general, the protective effect of doing strenuous sports can be observed in all income-age population segments, although in case of subjects of age above 61 this effect is small or negligible (see Supplementary Figures S3 and S4).

Figure 2 also suggests that moderate physical activity and exercises have similar interactions to strenuous sports with respect to lifetime depression. Supplementary Table S22 compares effects of moderate physical activity, exercises, and sports in conjunction with household income. Results indicate that all three activity related variables have a protective effect in every income subgroup, however the extent of this effect varies based on the activity and the income subgroups. In the majority of income subgroups (low – very high) whether or not doing strenuous sports has the largest effect on the depression. Exercises matter the most in case of the extremes of income subgroups: very low (No: 2.12, Yes: 1.36) and very high (No: 0.76, Yes: 0.46), and have a relatively smaller effect in other subgroups.

**Household income (I.)**		**I.+ Moderate physical (II.A)**				**I.+ Exercises (II.B)**				**I.+ Sports (II.C)**			
				$CI_{95\%}$				$CI_{95\%}$				$CI_{95\%}$	
	CR-OR		CR-OR	Low	High		CR-OR	Low	High		CR-OR	Low	High
Very low	1.91	No	2.10	1.94	2.26	No	2.12	1.98	2.27	No	1.93	1.82	2.06
		Yes	1.50	1.38	1.62	Yes	1.36	1.24	1.49	Yes	1.22	0.95	1.56
Low	1.08	No	1.25	1.16	1.35	No	1.25	1.16	1.35	No	1.13	1.06	1.20
		Yes	0.92	0.85	0.99	Yes	0.89	0.82	0.97	Yes	0.66	0.53	0.83
Moderate	0.90	No	1.04	0.96	1.12	No	1.04	0.96	1.12	No	0.96	0.90	1.02
		Yes	0.79	0.73	0.86	Yes	0.80	0.74	0.87	Yes	0.62	0.51	0.74
High	0.63	No	0.72	0.66	0.78	No	0.80	0.73	0.89	No	0.70	0.65	0.75
		Yes	0.60	0.54	0.67	Yes	0.57	0.52	0.63	Yes	0.45	0.37	0.54
Very high	0.55	No	0.58	0.49	0.69	No	0.76	0.62	0.93	No	0.62	0.54	0.72
		Yes	0.53	0.43	0.65	Yes	0.46	0.38	0.55	Yes	0.33	0.23	0.47

Supplementary Table S22. Parametric interactions of Household income, Moderate physical activity, Exercises and Sports with respect to lifetime depression. CR-OR and $CI_{95\%}$ denotes the configuration relative odds ratio and its 95% confidence interval respectively.

Moderate physical activity has the smallest effect on depression compared to the other two activity variables in general based on the average difference between effect sizes of performing and not performing a physical activity (moderate physical: 0.27, exercises: 0.38, sports: 0.41). In addition, moderate physical activity has its largest effect in the very low income subgroup (No: 2.1, Yes:1.5), and in contrast with sports and exercises, its effect with respect to depression gradually diminishes in higher income subgroups (e.g. in case of the very high income subgroup No: 0.58, Yes: 0.53, see Supplementary Figure S5).

Structural interaction results also indicate that there is a moderate interaction between body fat and strenuous sports with respect to lifetime depression. Table 4 shows the parametric aspect of this interaction involving strenuous sports and exercises. In general, both physical activity types provide a protective effect with respect to lifetime depression, whereas higher body fat percentage presents a risk (OR: 1.56, high versus normal). Regarding the joint effects of body fat and physical activity descriptors, the protective effect of doing sports or exercises is larger in case of subjects with high body fat percentage (e.g. sports CR-OR No: 1.62, Yes: 0.72) than in case of subjects with normal body fat percentage (e.g. sports CR-OR No: 0.74, Yes: 0.49). Compared to strenuous sports, the protective effect of exercises is smaller in both the normal (CR-OR No: 0.9, Yes: 0.59) and high (CR-OR No: 1.56, Yes: 0.92) body fat subgroups (shown in Figure 3).

The analysis of structural interactions suggested that parental depression plays a role in several interaction patterns. The first pattern involves sex and risk taking whose joint effect sizes are displayed in Supplementary Table S23. Parental depression has a considerable main effect (OR: 2.84, present versus not present) with respect to lifetime depression. The effect of sex is also relatively large (OR: 1.67, female versus male) although according to previous results it is not directly relevant with respect to lifetime depression. Risk taking on the other hand, has a negligible individual effect (OR: 1.07, Yes versus No). Supplementary Figure S6 displays the joint effect of parental depression, sex and risk taking. Results indicate that risk taking has a small but detectable effect in all parental depression - sex configurations, specifically the ratio of subjects with lifetime depression is higher among subjects that are risk takers than among non risk takers. This effect is larger in subgroups with presence of parental depression. The two endpoints of Parental depression - Sex - Risk taking variable configurations are (1) risk taking female subjects with parental depression (CR-OR: 3.22) and (2) non risk taking male subjects with no parental depression (CR-OR: 0.54).

The second interaction pattern related to parental depression involves parental Alzheimer's and life stress, corresponding multivariate effect sizes are shown in Supplementary Table S24. The presence and severity of life stress (negative life events in

Parental depression (I.)		I. + Sex (II.)		CI$_{95\%}$		I. + II. + Risk taking (III.)		CI$_{95\%}$	
	CR-OR		CR-OR	Low	High		CR-OR	Low	High
No	0.35	Female	1.11	1.06	1.18	No	1.02	0.97	1.08
						Yes	1.27	1.17	1.38
		Male	0.50	0.47	0.53	No	0.54	0.51	0.58
						Yes	0.60	0.55	0.66
Yes	2.84	Female	3.06	2.83	3.30	No	2.83	2.59	3.09
						Yes	3.22	2.80	3.70
		Male	2.04	1.84	2.27	No	1.94	1.69	2.21
						Yes	2.12	1.80	2.51

Supplementary Table S23. Parametric interactions of Parental depression, Sex and Risk taking with respect to lifetime depression. CR-OR and CI$_{95\%}$ denotes the configuration relative odds ratio and its 95% confidence interval respectively.

the past 2 years) has a major effect on current depressive symptoms, but also affects lifetime depression (OR(moderate versus low): 1.53, OR(high versus low): 2.59). The individual effect of the presence of parental Alzheimer's disease on the other hand is negligible (OR: 1.06). However, in a multivariate context parental Alzheimer's has a detectable effect together with parental depression. When parental depression is not present then the presence of parental Alzheimer's results in a higher ratio of subjects with lifetime depression (CR-OR: 0.85) than in case of subjects with no occurrence of parental Alzheimer's (CR-OR: 0.57). Interestingly, this effect is reversed in case of subjects with parental depression (parental Alzheimer's present: 2.47, Not present: 2.76), see Supplementary Figure S7. Life stress modulates these effect sizes according to the severity of life stress factors. Low severity serves as a protective factor, whereas high severity results in increased risk with respect to lifetime depression in each of these population subgroups.

Parental depression (I.)		I. + Parental Alzheimer's (II.)		CI$_{95\%}$		I. + II. + Life stress (III.)		CI$_{95\%}$	
	CR-OR		CR-OR	CI-L	CI-H		CR-OR	CI-L	CI-H
No	0.35	No	0.57	0.54	0.61	Low	0.49	0.47	0.52
						Moderate	0.96	0.90	1.02
						High	1.78	1.65	1.92
		Yes	0.85	0.79	0.93	Low	0.64	0.57	0.72
						Moderate	1.01	0.89	1.14
						High	1.41	1.19	1.68
Yes	2.84	No	2.76	2.57	2.96	Low	1.93	1.74	2.15
						Moderate	2.74	2.45	3.07
						High	4.21	3.67	4.84
		Yes	2.47	2.16	2.83	Low	1.80	1.44	2.25
						Moderate	2.47	1.97	3.09
						High	3.83	3.00	4.90

Supplementary Table S24. Parametric interactions of Parental depression, Parental Alzheimer's and Life stress with respect to lifetime depression. CR-OR and CI$_{95\%}$ denotes the configuration relative odds ratio and its 95% confidence interval respectively.

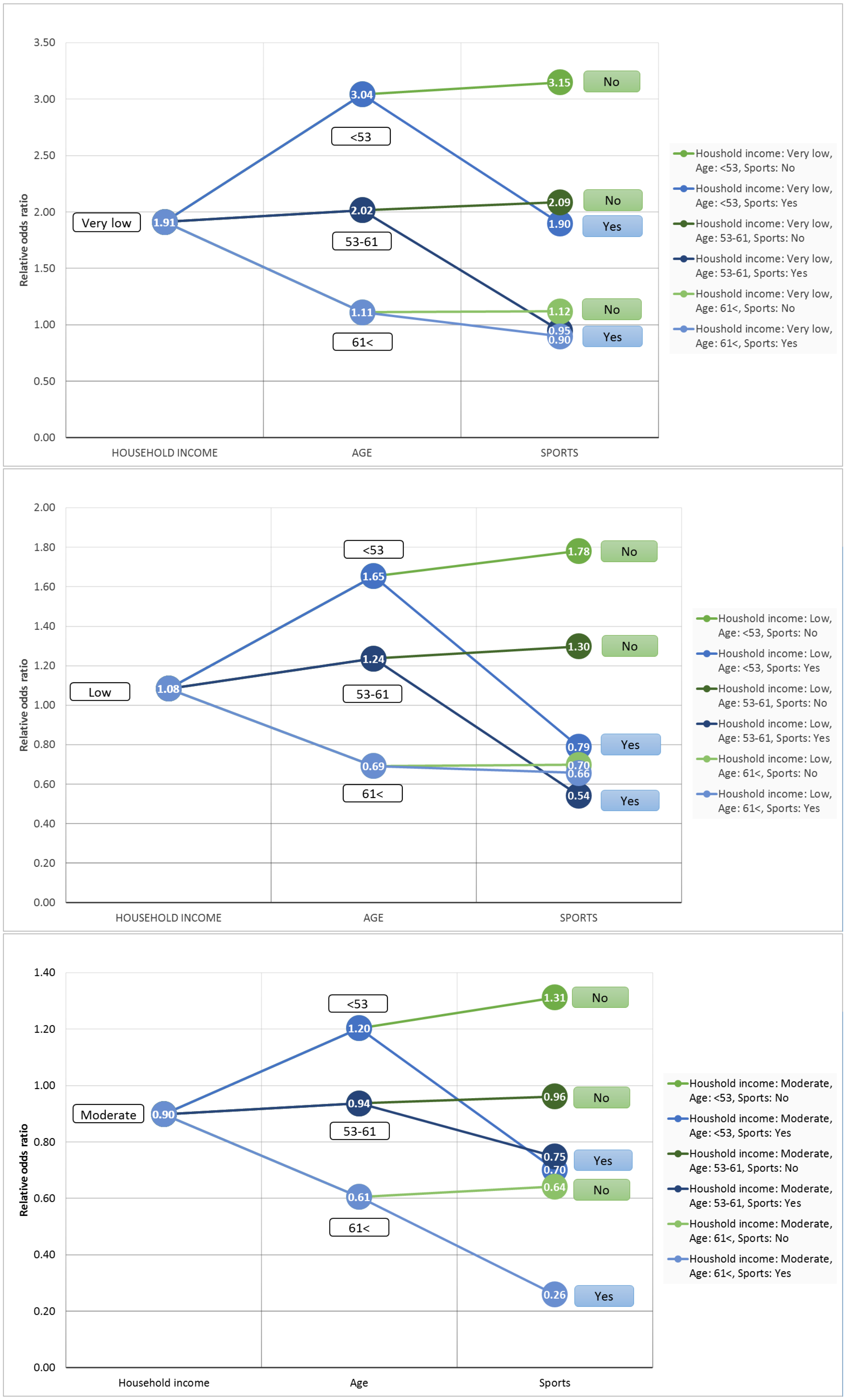

Supplementary Figure S3. **Parametric interactions of Household income, Age, and Sports with respect to lifetime depression.**

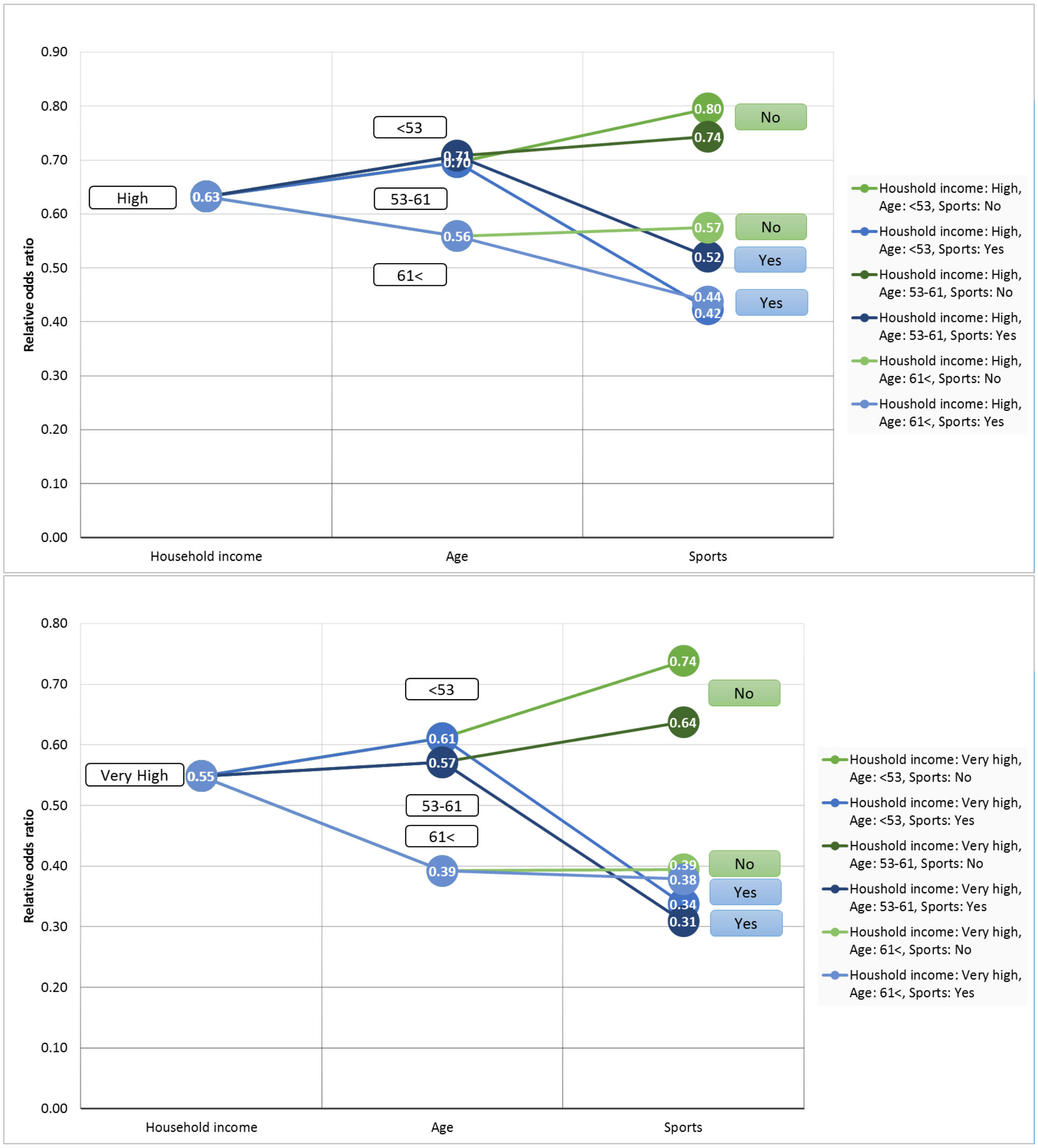

Supplementary Figure S4. **Parametric interactions of Household income, Age, and Sports with respect to lifetime depression.**

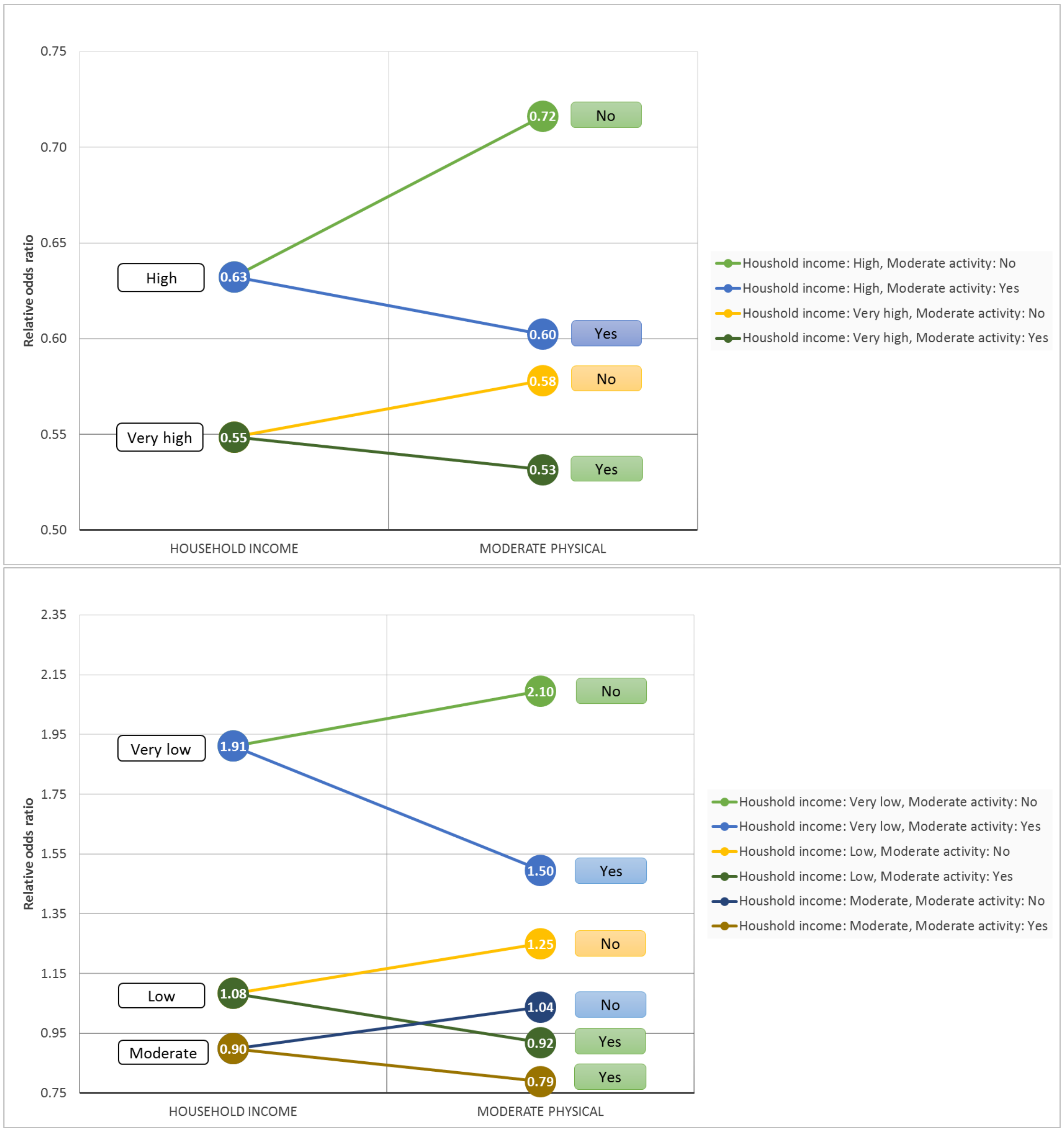

Supplementary Figure S5. Parametric interactions of Household income and Moderate physical activity with respect to lifetime depression.

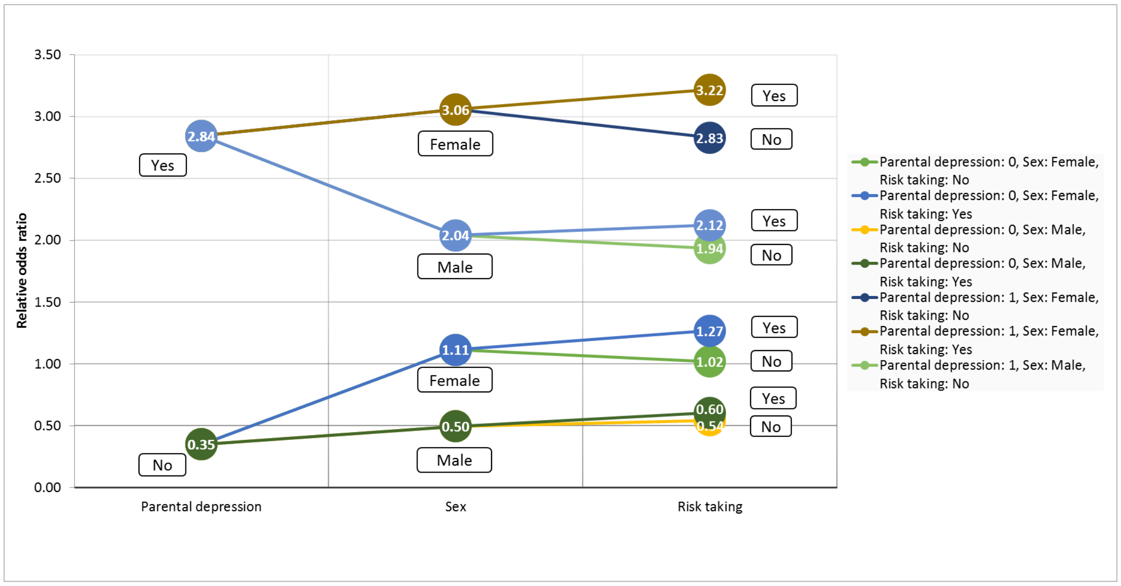

Supplementary Figure S6. **The effect of Parental depression, Sex and Risk taking on lifetime depression.**

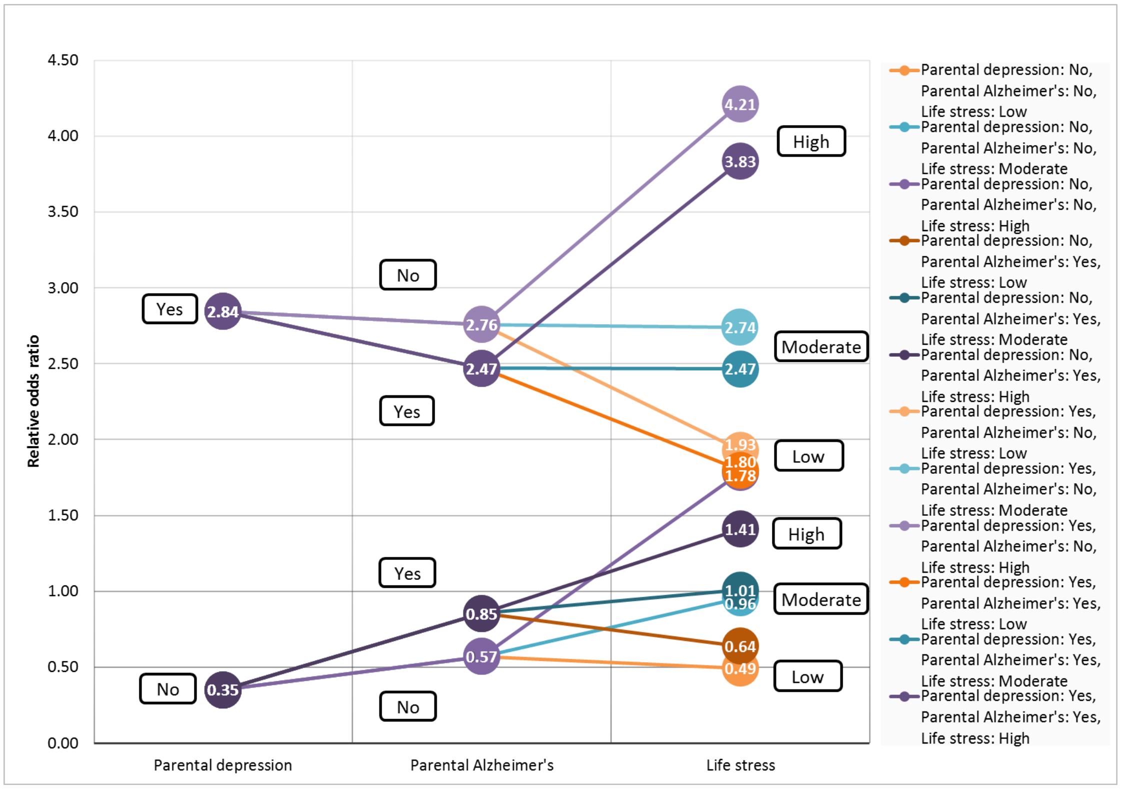

Supplementary Figure S7. **The effect of Parental depression, Parental Alzheimer's and Life stress on lifetime depression.**

The third interaction pattern consists of maternal smoking and life stress besides parental depression, their multivariate effect sizes are displayed in Supplementary Table S25. The individual effect of maternal smoking is small (OR: 1.15), increasing the risk of lifetime depression. In the context of parental depression the extent of this effect varies. In case of subjects with no parental depression the difference between the subgroups with maternal smoking (CR-OR: 0.92) and without maternal smoking (CR-OR: 0.62) is larger than in the presence of parental depression (CR-OR: 2.65 and 2.63 respectively), see Supplementary Figure S8. Similarly to the previous interaction pattern, life stress modifies these multivariate effects according to its severity. The only exception is in case of subjects with both parental depression and maternal smoking present, where moderate life stress produces the largest effect size (CR-OR: 4.24) both with respect to this subgroup and among all parental depression - maternal smoking - life stress variables configurations.

**Parental depression (I.)**		**I. + Maternal smoking (II.)**				**I. + II. + Life stress (III.)**			
				$CI_{95\%}$				$CI_{95\%}$	
	CR-OR		CR-OR	Low	High		CR-OR	Low	High
No	0.35	No	0.62	0.59	0.66	Low	0.50	0.47	0.54
						Moderate	0.66	0.61	0.72
						High	0.93	0.87	1.00
		Yes	0.92	0.86	0.98	Low	1.05	0.95	1.15
						Moderate	1.64	1.51	1.79
						High	1.79	1.59	2.01
Yes	2.84	No	2.63	2.43	2.84	Low	1.83	1.63	2.06
						Moderate	2.02	1.72	2.37
						High	2.70	2.38	3.06
		Yes	2.65	2.39	2.93	Low	2.60	2.19	3.08
						Moderate	4.24	3.64	4.95
						High	3.88	3.19	4.71

Supplementary Table S25. Parametric interactions of Parental depression, Maternal smoking and Life stress with respect to lifetime depression. CR-OR and $CI_{95\%}$ denotes the configuration relative odds ratio and its 95% confidence interval respectively.

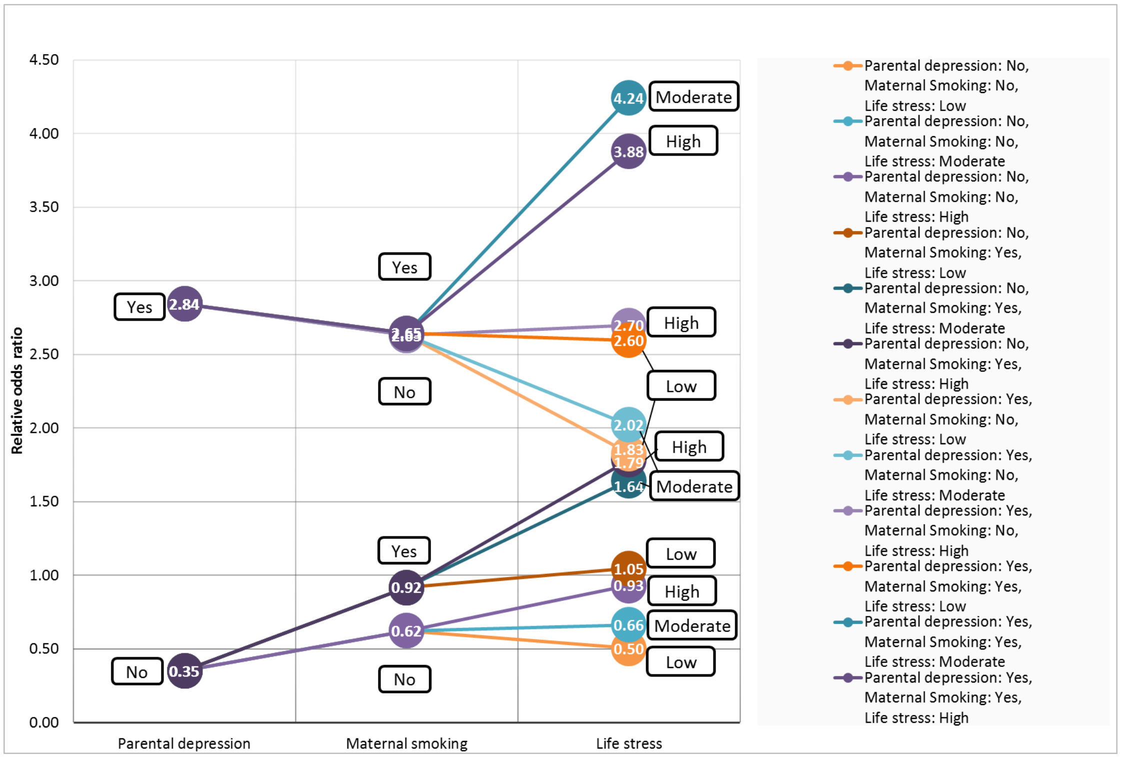

Supplementary Figure S8. The effect of Parental depression, Maternal smoking and Life stress on lifetime depression.

Finally, Figure 2 also indicated that qualification has a weak interaction with body fat, which proved to be insignificant from parametric perspective. However, further analysis revealed that qualification plays a role in a higher-order interaction with neuroticism and parental depression (see Supplementary Table S26). Individually, qualification has only a minor effect with respect to lifetime depression (OR(High versus Low): 1.10). In a multivariate context, depending on the neuroticism - parental depression configuration, different effects can be observed (see Supplementary Figure S9). In case of subjects with low or medium neuroticism trait scores and known parental depression, higher qualification serves as a minor protective factor (e.g. CR-OR(Neuroticism: Low| Parental depression: Yes|Qualification: High) = 0.77 versus CR-OR(...|...|Qualification: Low)= 0.88). In subgroups with no parental depression this effect is not observable. On the other hand, in case of subjects with high neuroticism score, qualification serves as a minor risk factor (e.g. CR-OR(Neuroticism: High| Parental depression: Yes|Qualification: High)= 6.14 versus CR-OR(...|...|Qualification: Low)= 5.81).

**Neuroticism (I.)**		**I. + Parental depression (II.)**				**I. + II. + Qualification (III.)**			
				$CI_{95\%}$				$CI_{95\%}$	
	CR-OR		CR-OR	Low	High		CR-OR	Low	High
Low	0.17	No	0.16	0.15	0.17	Low	0.24	0.22	0.27
						High	0.24	0.22	0.26
		Yes	0.82	0.72	0.95	Low	0.88	0.73	1.06
						High	0.77	0.63	0.95
Medium	1.25	No	1.08	1.02	1.16	Low	1.07	0.98	1.17
						High	1.08	1.00	1.17
		Yes	2.33	2.06	2.64	Low	2.37	1.99	2.83
						High	2.23	1.88	2.65
High	5.6	No	3.93	3.72	4.15	Low	2.97	2.75	3.20
						High	3.44	3.23	3.67
		Yes	6.37	5.85	6.94	Low	5.81	5.12	6.59
						High	6.14	5.5	6.86

Supplementary Table S26. Parametric interactions of Neuroticism, Parental depression, and Qualification with respect to lifetime depression. CR-OR and $CI_{95\%}$ denotes the configuration relative odds ratio and its 95% confidence interval respectively.

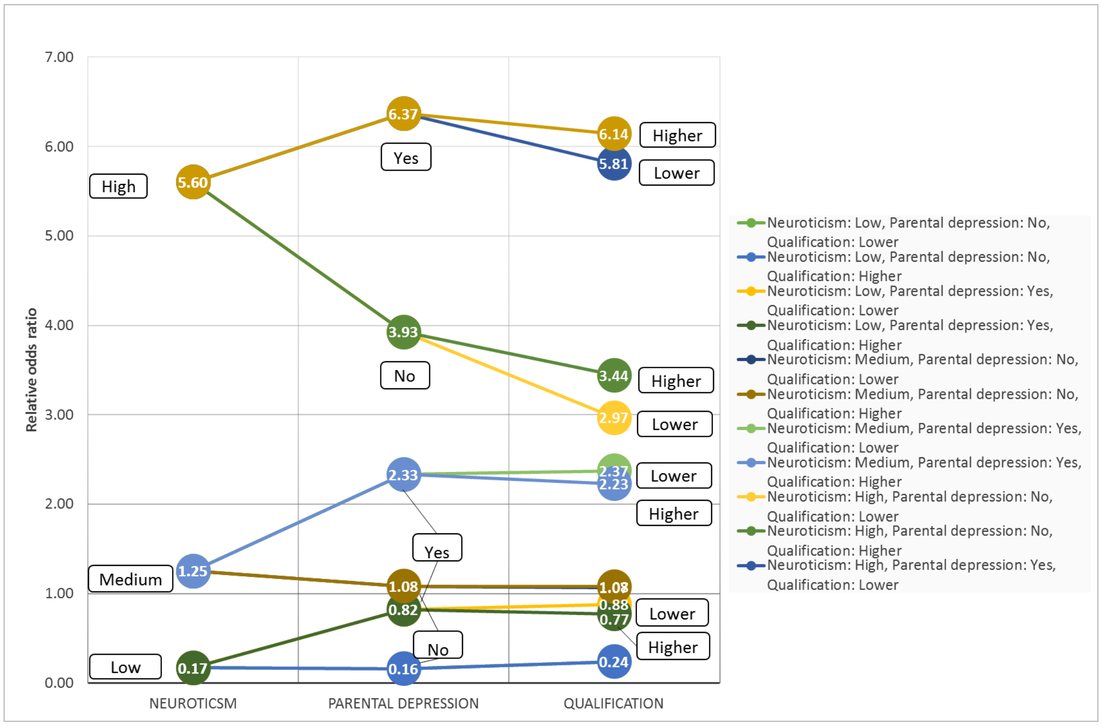

Supplementary Figure S9. **The effect of Neuroticism, Parental depression, and Qualification on lifetime depression.**
